# Plant transcriptome analysis reveals specific molecular interactions between alfalfa and its rhizobial symbionts below the species level

**DOI:** 10.1186/s12870-020-02503-3

**Published:** 2020-06-26

**Authors:** Wenjuan Kang, Zhehao Jiang, Yonggang Chen, Fang Wu, Chang Liu, Haifang Wang, Shangli Shi, Xue-Xian Zhang

**Affiliations:** 1 College of Grassland Science, Gansu Agricultural University, Lanzhou, 730070 China; 2 School of Natural and Computational Sciences, Massey University at Albany, Auckland, 0745 New Zealand; 3 College of Life Science and Technology, Gansu Agricultural University, Lanzhou, 730070 China; 4 Key Laboratory of Grassland Ecosystem of the Chinese Ministry of Education, Lanzhou, 730070 China

**Keywords:** Transcriptome, *Rhizobium*-legume symbiosis, Alfalfa cultivar, *Rhizobium*, Biotype

## Abstract

**Background:**

Leguminous plants alter patterns of gene expression in response to symbiotic colonization and infection by their cognate rhizobial bacteria, but the extent of the transcriptomic response has rarely been examined below the species level. Here we describe the identification of 12 rhizobial biotypes of *Ensifer meliloti*, which form nitrogen-fixing nodules in the roots of alfalfa (*Medicago sativa* L.), followed by a comparative RNA-seq analysis of four alfalfa cultivars each inoculated with two *E. meliloti* strains varying in symbiotic performance and phylogenetic relatedness.

**Results:**

Rhizobial biotypes were identified on the basis of their symbiotic performance, particularly shoot dry weight. Differentially expressed genes (DEGs) and metabolic pathways were determined by comparing the RNA-seq data with that of the uninoculated control plant. Significant differences were found between DEGs generated in each cultivar with the inoculation of two rhizobial strains in comparison (*P* < 0.01). A total of 8111 genes was differentially expressed, representing ~ 17.1% of the *M. sativa* genome*.* The proportion of DEGs ranges from 0.5 to 12.2% for each alfalfa cultivar. Interestingly, genes with predicted roles in flavonoid biosynthesis and plant-pathogen interaction (NBS-LRR) were identified as the most significant DEGs. Other DEGs include Medsa002106 and genes encoding nodulins and NCR peptides whose expression is specifically induced during the development of nitrogen-fixing nodules. More importantly, strong significant positive correlations were observed between plant transcriptomes (DEGs and KEGG pathways) and phylogenetic distances between the two rhizobial inoculants.

**Conclusions:**

Alfalfa expresses significantly distinct sets of genes in response to infection by different rhizobial strains at the below-species levels (i.e. biotype or strain). Candidate genes underlying the specific interactions include Medsa002106 and those encoding nodulins and NCR peptides and proteins in the NBS-LRR family.

## Background

The initiation and development of legume-rhizobial symbiosis require numerous signal exchanges and regulatory approaches between host plants and microbial symbionts. The plant-derived flavonoids induce expression of a LysR-type regulator NodD, which activates transcription of the structural nodulation (*nod*, *nol*, *noe*) genes in rhizobia [1]. These nodulation genes are responsible for the biosynthesis and exporting of nodulation factors (NFs). Next, the bacteria-derived NFs are detected by the lysin Motif Receptor-like Kinase (LysM-RLK) in plant, causing nodule organogenesis and rhizobial infection into plant roots through infection threads [1]. After being released from the infection thread, rhizobial cells are encompassed in a plant-derived membrane structure and undergo differentiation into bacteroids. This eventually leads to the formation of symbiosomes wherein atmospheric nitrogen is converted into ammonium [2, 3]. The terminal bacteroid differentiation process is governed by nodule-specific peptides such as small nodulin acidic RNA binding proteins (SNARPs), nodule-specific cysteine-rich peptides (NCRs) and glycine-rich proteins (GRPs) [4, 5]. They are thus required for the establishment of highly efficient nitrogen-fixation symbiotic systems.

The symbiotic interactions between legumes and rhizobia are highly specific and can occur at and below the levels of species [6]. A well-studied example is legumes in the genus of *Medicago* and their symbionts *Ensifer meliloti*. *M. laciniate* and *M. rigiduloides* form efficient nitrogen-fixing nodules with *E. meliloti* bv. *medicaginis* and *E. meliloti* sv. *rigiduloides*, respectively [7]. As mentioned above, host specificity is largely determined by flavonoids and NFs produced by plant and rhizobia, respectively [8, 9]. The chemical structure of a NF is crucial for specific recognition by a particular host plant. All NFs possess a conserved core structure but vary in chemical modifications that are mediated by specific rhizobial nodulation proteins [7, 10]. However, a single bacterial strain can produce various forms of NFs [11]. On the other hand, *Medicago* genomes encode more than 700 NCR genes, which are specifically expressed in the nodule. The diverse NCR peptide repertoire confers on host plant the potential to recognize and manipulate rhizobial infection in a strain-specific manner [12, 13]. Moreover, plant innate immunity is involved in controlling the early stages of bacterial infection, and also determines plant cell differentiation at the later stage of the symbiotic process [14]. It is thus highly plausible to speculate that leguminous plants display distinct patterns of gene expression in response to infection by different rhizobial strains below the species levels such as biovar (or symbiovar), ecotype or strain.

Next-generation sequencing approaches have provided insights into the global responses of plant cells to bacterial infection in certain model taxa [15, 16]. More specifically, RNA-seq transcriptional profiling allows the detection of all genes that are differentially expressed during symbiosis. Recent studies identified symbiotic traits such as oxygen response [17], plant immunity [18], and the production of plant hormones [16, 19–21] and secondary metabolites [22–24]. Distinct transcriptomic responses were evidenced for soybean (*Glycine max*) inoculated with rhizobial strains belonging to two different genus, *Bradyrhizobium japonicum* and *Sinorhizobium (Ensifer) fredii* [25]. Moreover, transcriptomic profiles of *Lotus japonicus* were compared upon inoculation with compatible rhizobial, non-adapted rhizobial and pathogenic bacterial strains, and the data revealed no general early defense-like responses evoked by compatible rhizobia [26].

Rhizobia are normally classified at the species level, but further assignment below the species level is required to better understand the specific rhizobium-legume interactions [27]. Symbiovar was previously proposed to reflect the capability of a rhizobial strain to nodulate legumes regardless of the species to which they belong [27–31]. Biotype affiliation of a particular rhizobial strain is also useful in agriculture, as multiple cultivars are involved for almost every leguminous crop. While the concept of biotype classification has been widely adopted, it remains elusive how host plants genetically respond to rhizobial infections at the below-species level.

Here we report a comparative RNA-seq analysis of four alfalfa cultivars each being subjected to infections by two different rhizobial strains plus an uninoculated control. Our work began with symbiotic characterization of 32 rhizobial isolates, which led to the identification of 12 unique biotypes. The knowledge was then used to design a large-scale plant transcriptome experiment that involved four alfalfa cultivars and six rhizobial biotypes. Results of transcriptomic analysis consistently indicate that legume plants express significantly different sets of genes in response to rhizobial infection at the biotype level. Molecular mechanisms underlying the specific legume-rhizobial interactions will be discussed with predicted functions of the differentially expressed genes and metabolic pathways.

## Results

### Assessing the symbiotic efficiency of alfalfa-associated rhizobia

**Fig. 1:**
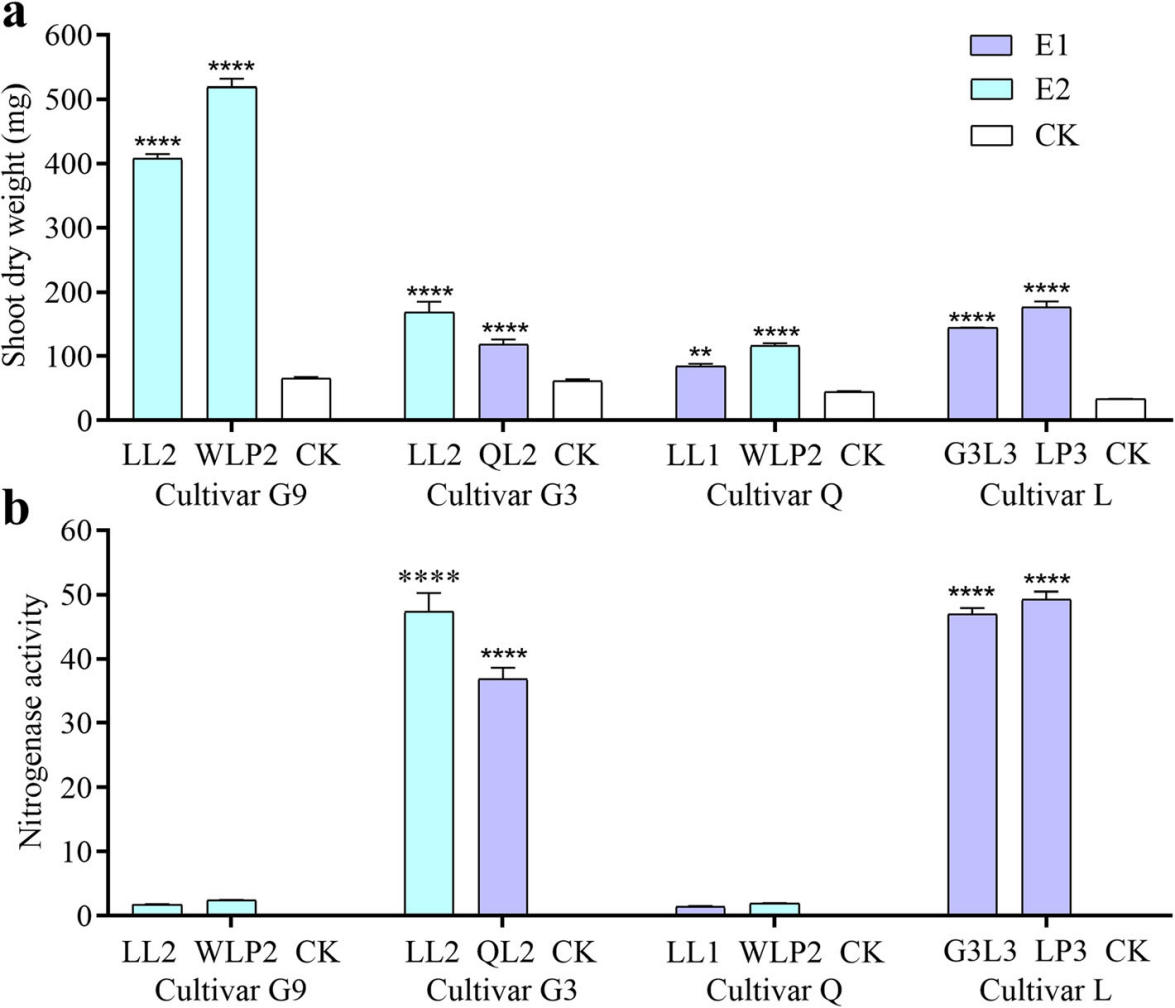
Symbiotic performance of *E. meliloti* strains selected for plant transcriptome analysis. Shoot dry weights (**a**) and nitrogenase activities (**b**) were measured for four *M. sativa* cultivars: G9, Gannong No. 9; G3, Gannong No. 3; Q, Qingshui; L, Longzhong. Data are means and standard errors of four biological replicates. Nitrogenase activities were expressed as μmol·g^− 1^·h^− 1^. Level of significance was indicated by either two stars (*P* < 0.01) or four stars (*P* < 0.0001) above the bar. The Student’s *t*-test was performed in comparison with the uninoculated controls (CK). Effective one- and two-cultivar specificity biotypes (E1 vs. E2) were distinguished by two different colors

Phylogenetic relationships of the 32 rhizobial isolates (listed in Additional file 1) were analyzed on the basis of 16S rRNA gene sequences. Results showed that they were all closely related to the type strain of *E. meliloti* (Additional file 2). Next, symbiotic performance was determined on five *M. sativa* cultivars in terms of 14 parameters, and data are provided for each of the five plant cultivars in Additional files 3, 4, 5, 6 and 7 respectively. Results of shoot dry weight (SDW) and nitrogenase activity are summarized in Fig. 1 for strains selected for plant transcriptome analysis (see below). Consistent with our expectation, large variations were observed among different rhizobial strains and also among different alfalfa cultivars, implicating specific plant-bacterial interactions. Using SDW data as an example, alfalfa cultivar G9 produced the highest scores when compared with uninoculated control (Fig. 1). Significantly higher average SDWs were found for three rhizobial strains LL11 (639.67 mg), WLP2 (519.33 mg) and LL2 (407.53 mg) (Additional file 3). Intriguingly, nine rhizobial strains produced significantly lower SDWs, suggesting that rhizobial infection can be detrimental for incompatible plant cultivars. On the other hand, for a single rhizobial strain WLP2 significantly higher SDWs were detected with plant cultivars G9 (519.33 mg) and Q (116.23 mg), but not with cultivars L (42.97 mg), WL (124.37 mg), and G3 (44.4 mg) when compared with the uninoculated control (61.4 mg). Similar phenomenon was found with the nitrogenase activities (Fig. 1, Additional files 3, 4, 5, 6 and 7). Together, the symbiosis data consistently indicate that the alfalfa-rhizobial interactions are highly specific at the below-species levels.

**Fig. 2:**
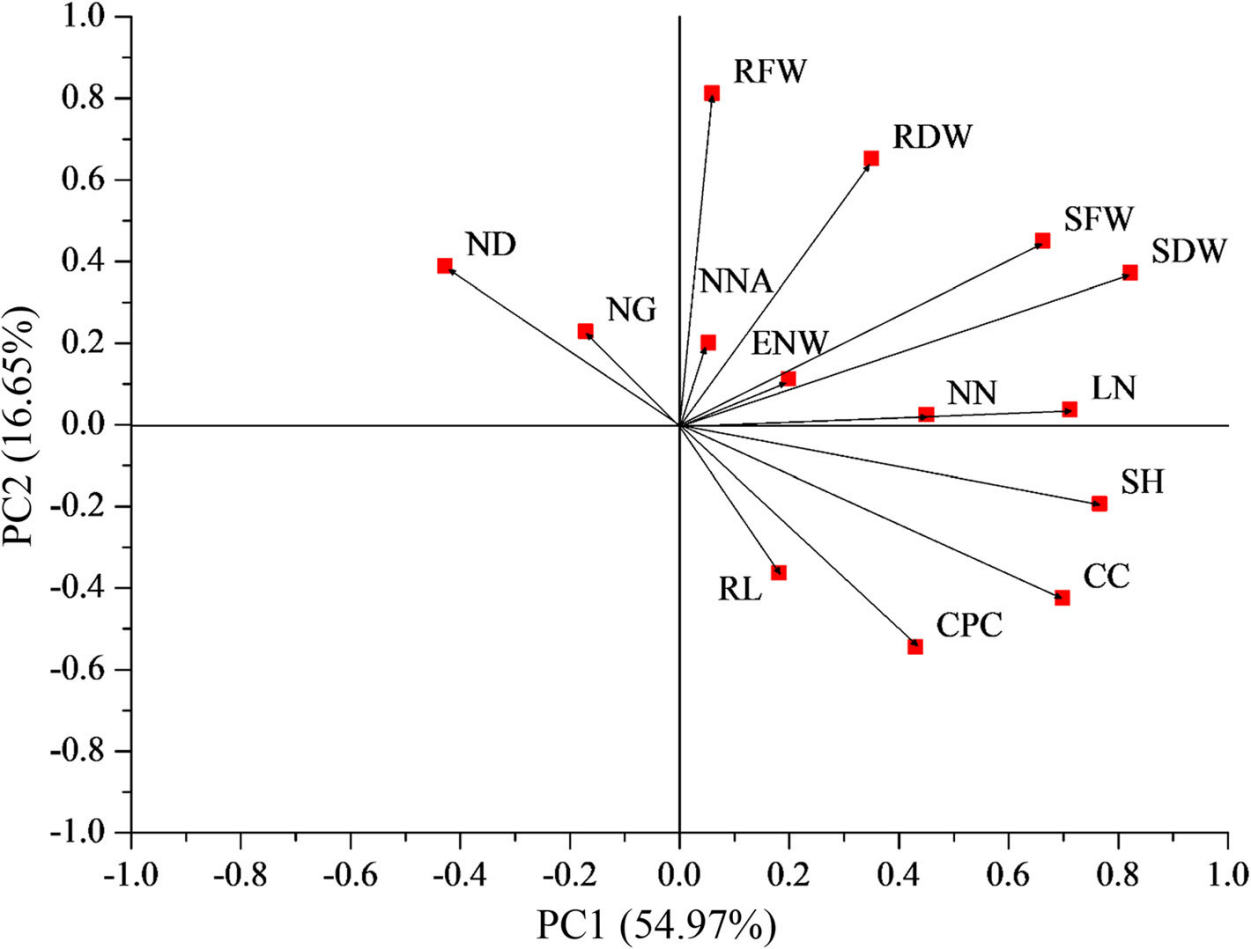
Principal component analysis of alfalfa-rhizobia interaction on the basis of 14 symbiotic parameters. NN, nodule number; ENW, effective nodule weight; ND, nodule diameter; NG, nodule grade; NNA, nodule nitrogenase activity; LN, leaf number; SH, shoot height; RL, root length; SFW, shoot fresh weight; RFW, root fresh weight; SDW, shoot dry weight; RDW, root dry weight; CC, chlorophyll content; CPC, crude protein content

Next, the symbiotic data of 14 parameters were subjected to principal component analysis (PCA) whereby the relative importance of each parameter was assessed. Results suggest that plant SDW contributed the most to the observed variations, whereas nitrogenase activities had the least effects on symbiotic diversity (Fig. 2). Furthermore, there was a weak positive correlation between SDW values and nitrogenase activities (*R* = 0.1653, *P* = 0.0338). SDW was thus selected as the representative parameter for estimating rhizobial symbiotic efficiency.

### Biotype classification of the *E. meliloti* isolates

The 32 isolates were arbitrarily assigned to three symbiotic categories (effective, E; inhibitory, I; noneffective, O), which represent significantly higher, lower or no difference (*P* < 0.05) in terms of plant SDW when compared with the uninoculated controls (Additional file 8). The symbiotic category for each rhizobium was then combined with the alfalfa cultivar listed in the order of G3, G9, L, Q and WL. This resulted in a total of 12 symbiotic patterns (i.e. biotypes) for the 32 *E. meliloti* strains on five alfalfa cultivars (Table 1). Each biotype has a unique symbiotic specificity with the tested alfalfa cultivars. For example, biotype I, II, III and IV display an effective one-cultivar specificity with alfalfa cultivar G3, G9, L and Q, respectively. However, biotypes IX, X and XI show an inhibitory one-cultivar specificity with alfalfa cultivar G9, Q and WL, respectively. Effective two-cultivars specificity was observed for biotypes V (alfalfa cultivars G3 and G9) and VI (alfalfa cultivars G9 and Q), while inhibitory two-cultivars specificity was observed for biotype XII (cultivars Q and WL). Additionally, biotype VIII didn’t form effective symbiosis with any of the alfalfa cultivars.

**Table 1:** Biotype classification of 32 *E. meliloti* isolates on the basis of their symbiotic profiles

Strain name^**a**^	Symbiotic pattern^**b**^	Ratio of E:O:I	Biotype^**c**^	Specificity^**d**^
QL2, WLG1	EOOOO	1:4:0	I	E1
LL11	OEOOO	1:4:0	II	E1
G3L3, LP3	OOEOO	1:4:0	III	E1
LL1	OOOEO	1:4:0	IV	E1
LL2	EEOOO	2:3:0	V	E2
WLP2	OEOEO	2:3:0	VI	E2
G9L3, G9L8	OIOEO	1:3:1	VII	E1I1
G3L2, G3L6, G3L8, G3T2, G9L4, G9L6, LL5, LL6, LL7	OOOOO	0:5:0	VIII	N
G3L4, G3L7, G3L10, G3L12, G3L13, G9L5, LL10	OIOOO	0:4:1	IX	I1
G3L9	OOOIO	0:4:1	X	I1
G3L5, LL8, QL4, QL5	OOOOI	0:4:1	XI	I1
G9L7	OOOII	0:3:2	XII	I2

^a^A prefix was assigned to inform plant cultivar from which the strains was originally isolated: G3 for Gannong No. 3; G9, Gannong No. 9; L, Longzhong; Q, Qingshui; WL, WL168HQ

^b^The symbiotic pattern was obtained by combining the marked symbiotic efficiency of each strain in the order of cultivar G3, G9, L, Q and WL. Letter E (effective), O (noneffective), or I (inhibitory) indicates shoot dry weight value was significantly higher, not significantly different, or significantly lower (*P* < 0.05) when compared with uninoculated plants, respectively (Additional files 8)

^c^Roman numerals refer to rhizobial biotypes for strains determined based on the symbiotic pattern

^d^E1 Effective one-cultivar specific biotype, E2 Effective two-cultivars specific biotype, E1I1 Effective one-cultivar and inhibitive one-cultivar specific biotype, N Non-specific biotype, I1 Inhibitory one-cultivar specific biotype, I2 Inhibitory two-cultivars specific biotype

### RNA-seq experimental design

**Fig. 3:**
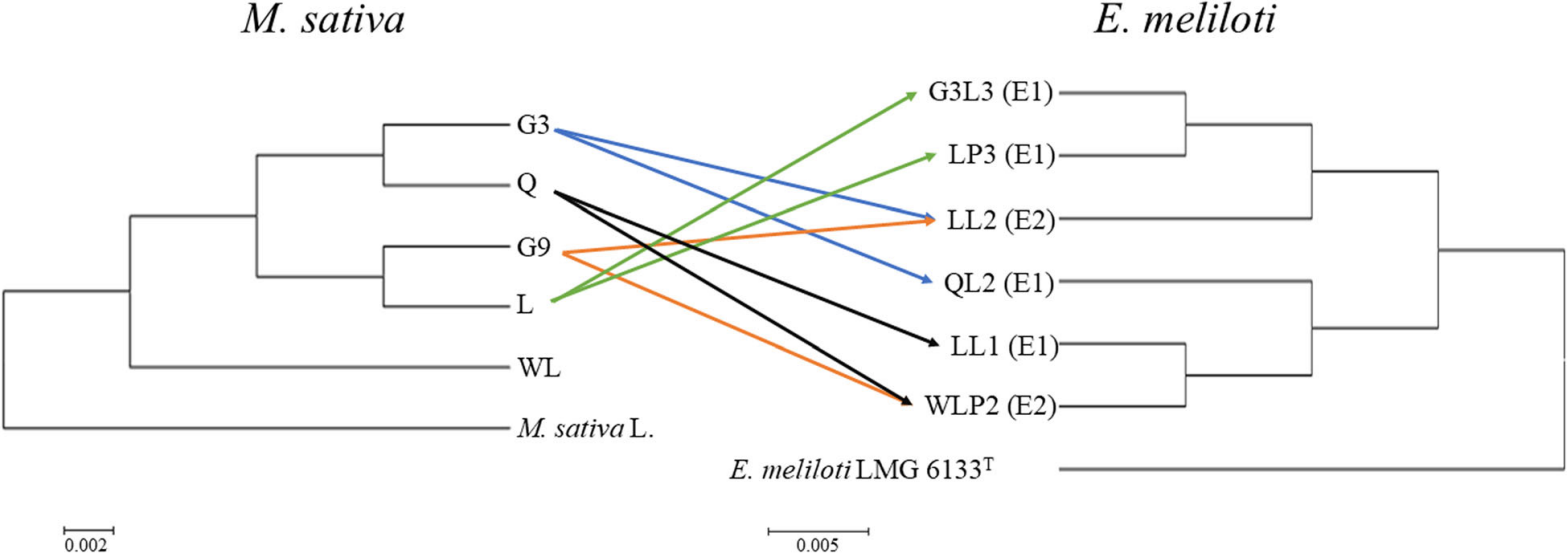
Experimental design of the plant transcriptomic analysis. A Neighbor-Joining tree is shown on the left for five alfalfa (*Medicago sativa*) cultivars Gannong No. 3 (G3), Gannong No. 9 (G9), Longzhong (L), Qingshui (Q), and WL168HQ (WL). Phylogenetic relatedness among the six rhizobial strains is shown on the right. All six rhizobial strains can form effective nodules on the related cultivars, and their effective one- or two-cultivar specificity type (E1 or E2) are indicated in parenthesis

The results of symbiotic assays described above led us to hypothesize that (i) transcriptomes of an alfalfa cultivar differ significantly in response to rhizobial infections at the below-species levels; (ii) effective one-cultivar specific biotype (E1) and effective two-cultivars specific biotype (E2) cause different patterns of global gene expression on the same host plant. To test these hypotheses, transcriptome analysis was performed with six rhizobial strains and four alfalfa cultivars, exhibiting 12 unique symbiotic interactions (Fig. 3). Additionally, uninoculated controls were set up for each of the four plant cultivars. G3-QL2, L-LP3, L-G3L3, and Q-LL1 represented interactions between an E1 strain and its specific cultivar, whereas G3-LL2, G9-LL2, G9-WLP2, and Q-WLP2 constituted interactions between an E2 strain and two specific cultivars (Fig. 3, Table 1). In terms of biotype, one alfalfa cultivar (L) was inoculated with two rhizobial strains (G3L3 and LP3) of the same biotype, whereas all other three alfalfa cultivars were inoculated with rhizobial strains of different biotypes.

Figure 3 shows phylogenetic relationships among the five alfalfa cultivars, and separately, the six rhizobial strains together with the reference species. The plant phylogenic tree was constructed using concatenated sequence of four housekeeping genes (*matK1*, *matK2*, *matK3*, and *rbcL*) with 97% or greater sequence similarity. Genetic relatedness of the six bacterial strains was estimated using 16S rRNA gene sequences with the type strain *E. meliloti* LMG 6133^T^ as the outgroup. Of note, alfalfa cultivar WL was excluded from the RNA-seq analysis as it does not support efficient symbiosis with any of the 32 rhizobial strains in terms of significantly increased SDW values.

### RNA-seq read statistics and function annotation

A total of 95,120 unigenes were generated and used for similarity searching and function annotation against the commonly used databases (Additional file 9). Length distribution of the assembled transcripts and taxonomic source of Basic Local Alignment Search Tool (BLAST) matches for *M. sativa* unigenes were presented in Additional file 10. A total of 67,815 unigenes were grouped into specialized GO (gene ontology) functional categories according to the ontologies of biological process, cellular component and molecular function using Blast2GO software v2.3.5 (Additional file 11a). A total of 20,444 assembled unigenes were correlated with 127 KEGG (Kyoto Encyclopedia of Genes and Genomes) pathways and a 19-pathway hierarchy 2 (Additional file 11b). The functional annotation and pathway assignment based on GO and KEGG revealed high diversity of functional proteins and metabolic pathways in *M. sativa* transcriptomes.

### Variation analysis of differentially expressed genes (DEGs)

**Fig. 4:**
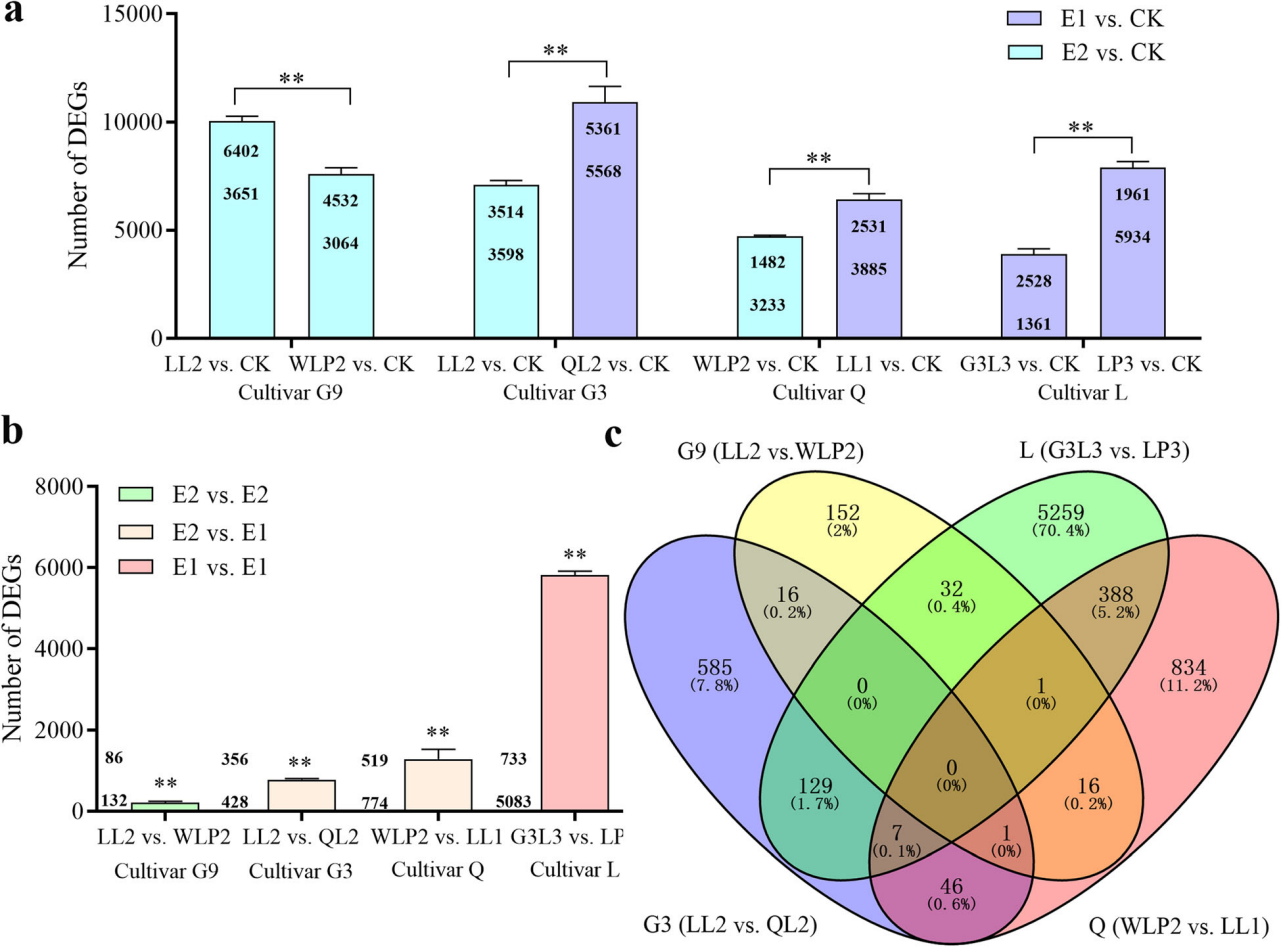
Variation of DEGs for each alfalfa cultivar inoculated with two different rhizobial strains. **a** DEGs detected in biotype vs. CK comparisons. Number of upregulated (top) and downregulated (down) genes is indicated within each column. Data are means and standard errors of three biological replicates. **b** DEGs detected in biotype comparisons; **c** A Venn diagram of DEGs in biotype comparisons; **P < 0.01; E2, Effective two-cultivars specific biotype; E1, Effective one-cultivar specific biotype; CK, uninoculated control; G9, *M. sativa* cv. Gannong No. 9; G3, *M. sativa* cv. Gannong No. 3; Q, *M. sativa* cv. Qingshui; L, *M. sativa* cv. Longzhong

Genes with |log_2_ (fold change, FC) | ≥ 1 and false discovery rate (FDR, corrected-*p* value) < 0.05 were designated DEGs. DEGs were first generated for the eight rhizobial inoculated plants relative to uninoculated controls. Results revealed significant difference of DEGs numbers when G9 was inoculated with two E2 rhizobial strains (*q* < 0.01). The same was found for cultivar L inoculated with two E1 strains (*q* < 0.01). For the G3 and Q cultivars, it appears that inoculation with an E1 strain caused significantly higher DEGs than an E2 strain (*q* < 0.01). When an E2 strain (LL2 or WLP2) was inoculated to two different plant cultivars the numbers of DEGs were significantly higher in G9 than in G3 (Fig. 4a). The data thus indicated that cultivar G9 displayed a larger transcriptomic response to E2 rhizobial infections than G3 and Q cultivars. The numbers of common genes across different treatments are presented in Additional file 12. The data showed that 0.23% of genes in the *Medicago* genome are involved in the formation of efficient nitrogen-fixing nodules.

Additionally, DEGs were also generated for each cultivar inoculated with the two rhizobial strains at the variation level of *q* < 0.01 (Fig. 4a). A total of 8111 genes were differentially expressed, representing 17.1% of the entire set of genes in the *M. truncatula* genome. Results indicate a biotype-specific transcriptomic response for each of the four alfalfa cultivars (Fig. 4b). The largest effects were detected for cultivar L inoculated with two E1 strains with 5816 DEGs (12.2%). Fewer DEGs were observed for cultivars Q (1293) and G3 (784) when they were inoculated with an E1- versus E2-type strain. Only 218 DEGs were detected for cultivar G9 inoculated with two E2 strains (Fig. 4b). The number of DEGs differed significantly among the four cultivars (*q* < 0.01). No common DEGs were found for the four cultivars (Fig. 4c). However, one DEG (Medsa002106) was shared by three cultivars (G9, Q and G3), suggesting the potential role of this gene in the specific recognition of rhizobia at the biotype level.

### GO terms and KEGG pathways enriched by DEGs for each alfalfa cultivar inoculated with two rhizobial strains

**Fig. 5:**
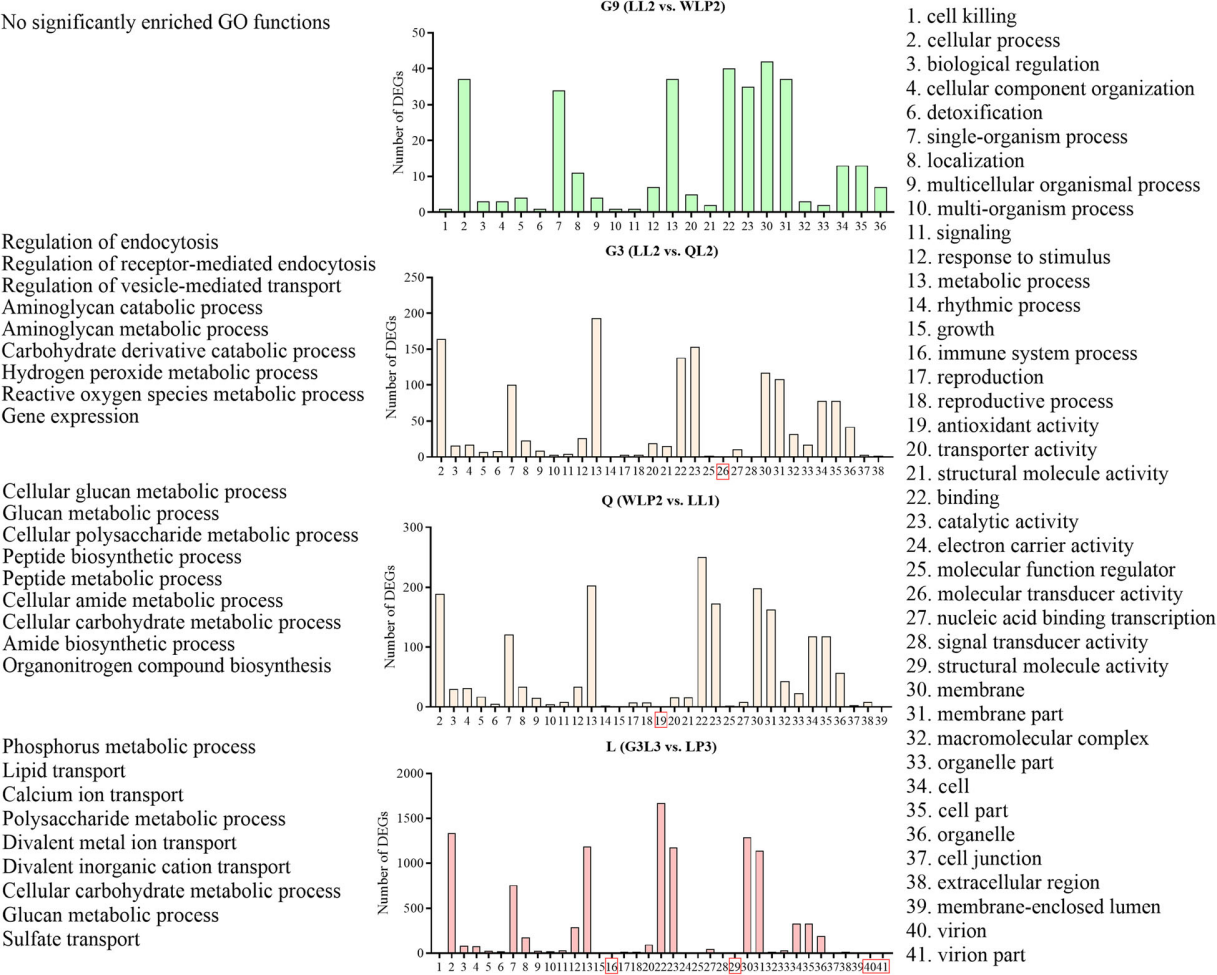
Functional annotation of the differentially expressed genes. The analysis involved 41 GO functions in three categories shown on the right: biological process (1–18), cellular component (19–29) and molecular function (30–41) with 21 GO terms (2–13, 20, 22, 23, 30–36) enriched by DEGs common for four cultivars. Terms labelled with red boxes were exclusively enriched in only one comparison. Top nine GO terms significantly enriched (*q*-value < 0.05) are listed on the left. G9, *M. sativa* cv. Gannong No. 9; G3, *M. sativa* cv. Gannong No. 3; Q, *M. sativa* cv. Qingshui; L, *M. sativa* cv. Longzhong

The GO enrichment analysis identified 21 GO terms jointly enriched by DEGs for the four cultivars (Fig. 5). More GOs were revealed for the two E1-type strains in cultivar L when compared with the two E1/E2 treatments (cultivars G3 and Q). No GO function was significantly enriched for two E2 strains in cultivar G9 (*q*-value< 0.05). Specific for E1 versus E2 strains, metabolic genes involved in endocytosis, hydrogen peroxide and reactive oxygen species were significantly enriched in G3 (LL2 vs. QL2), whereas the metabolic processes of cellular polysaccharide, glucan, peptide, amide and carbohydrate were enriched in Q (WLP2 vs. LL1).

**Fig. 6:**
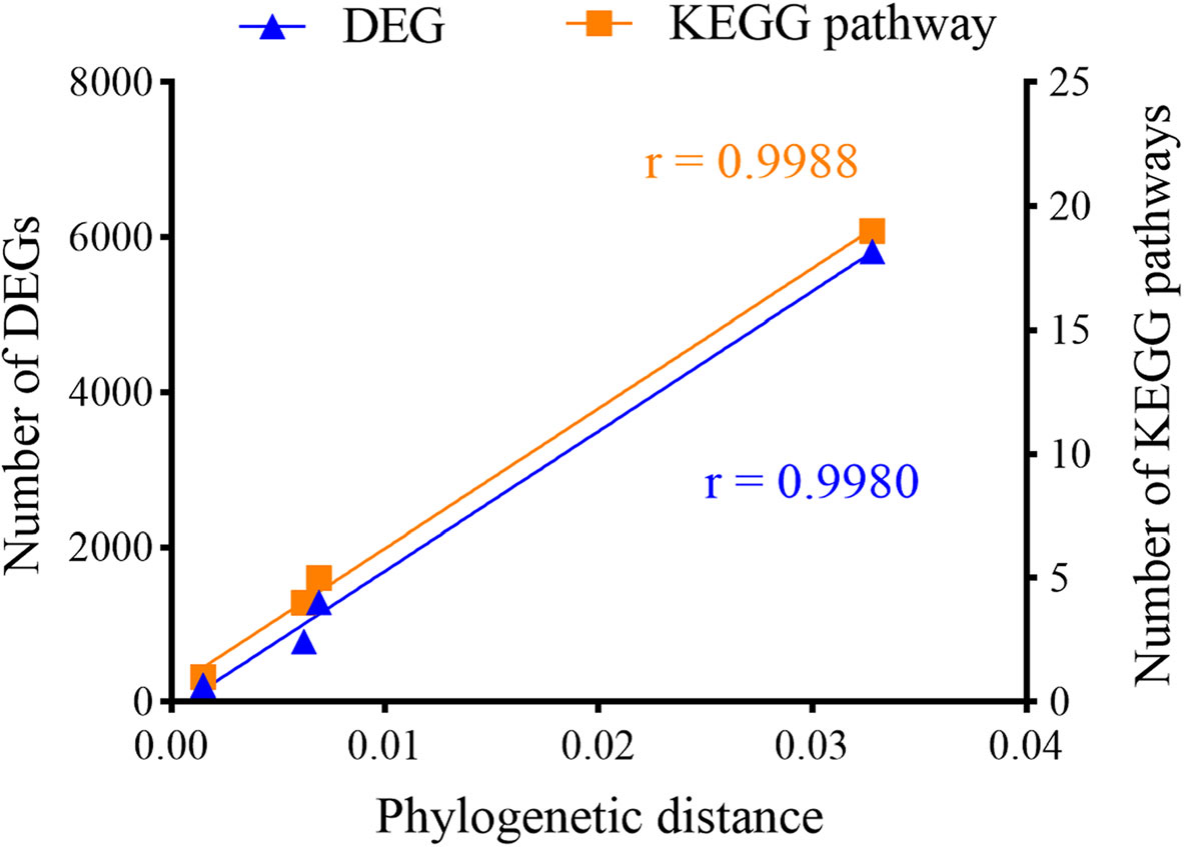
Correlation of plant transcriptomes with phylogenetic distances between the two bacterial inoculants in comparison. The plant transcriptomic responses are shown as the number of differentially expressed genes (DEGs) and the number of KEGG pathways. The pearson correlation coefficients (*r*) were determined using Graphpad Prism v 8.0 (GraphPad Software Inc., San Diego)

The KEGG pathways significantly enriched by DEGs from biotype comparisons (*q*-value < 0.05) were shown in Table 2. DEGs in G3 (LL2 vs. QL2) and Q (WLP2 vs. LL1) were associated with ribosome, valine, leucine and isoleucine biosynthesis, and terpenoid biosynthesis. However, flavonoid biosynthesis and plant-pathogen interaction were the two predominantly involved pathways for plant cultivar L (G3L3 vs. LP3). Significantly, there was a positive correlation between the number of DEGs and the phylogenetic distance between the two rhizobial strains in comparison (Fig. 6).

**Table 2:** KEGG pathway enrichment analysis of differentially expressed genes

Comparison ^**a**^	KEGG Pathway ^**b**^	*P-*value ^**c**^	*q-*value ^**c**^	Number of genes
G9 (LL2 vs. WLP2)	Oxidative phosphorylation	3.99E-03		4
G3 (LL2 vs. QL2)	Ribosome	1.17E-18	6.45E-17	52
	Diterpenoid biosynthesis	4.82E-03		4
	Phenylpropanoid biosynthesis	8.15E-03		11
	Valine, leucine and isoleucine biosynthesis	3.15E-02		2
Q (WLP2 vs. LL1)	Ribosome	1.60E-21	1.14E-19	74
	Sesquiterpenoid and triterpenoid biosynthesis	2.26E-03		4
	Circadian rhythm - plant	6.71E-03		5
	Valine, leucine and isoleucine biosynthesis	1.05E-02		3
	Amino sugar and nucleotide sugar metabolism	3.71E-02		10
L (G3L3 vs. LP3)	Flavonoid biosynthesis	3.72E-12	4.09E-10	25
	Plant-pathogen interaction	9.35E-11	5.14E-09	73
	Alpha-Linolenic acid metabolism	7.34E-09	2.69E-07	24
	Phenylpropanoid biosynthesis	3.33E-07	9.16E-06	47
	Stilbenoid, diarylheptanoid and gingerol biosynthesis	1.12E-06	2.47E-05	12
	Plant hormone signal transduction	1.95E-06	3.58E-05	44
	Circadian rhythm - plant	1.80E-05	2.83E-04	13
	Starch and sucrose metabolism	2.44E-05	3.36E-04	47
	Pentose and glucuronate interconversions	4.34E-05	5.31E-04	23
	ABC transporters	2.16E-04	2.38E-03	15
	Linoleic acid metabolism	1.18E-03	1.18E-02	11
	Carotenoid biosynthesis	2.71E-03	2.49E-02	10
	Terpenoid backbone biosynthesis	6.39E-03	5.41E-02	11
	Biosynthesis of unsaturated fatty acids	1.22E-02	9.01E-02	10
	Glucosinolate biosynthesis	1.23E-02	9.01E-02	2
	Zeatin biosynthesis	2.49E-02		5
	Phosphatidylinositol signaling system	3.56E-02		12
	Cyanoamino acid metabolism	3.64E-02		14
	Glycerophospholipid metabolism	3.79E-02		14

^a^Biotype comparison on alfalfa cultivars G9 (Gannong No. 9), G3 (Gannong No. 3), Q (Qingshui) and L (Longzhong)

^b^Only pathways with *p*-value or *q*-value < 0.05 were presented

^c^The *q-*value is a natural pFDR (positive false discovery rate) analogue to the *p*-value. KEGG pathways with *q*-value < 0.05 were defined as significantly enriched pathways

### Expression of nodule inception, leghemoglobin and glutamine synthetase genes

**Fig. 7:**
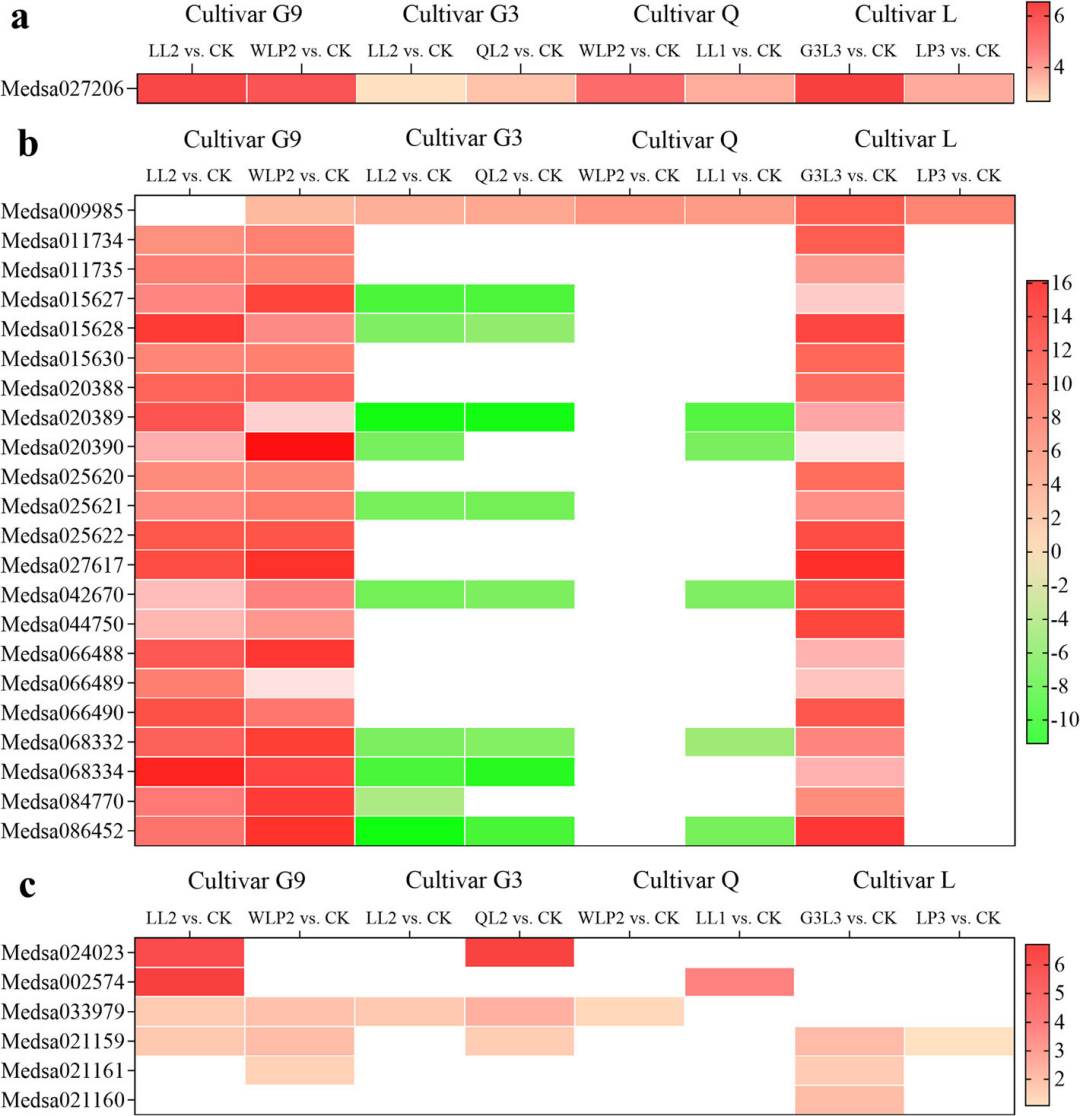
Expression of genes encoding nodule inception (**a**), leghemoglobin (**b**) and glutamine synthetase (**c**) in four alfalfa cultivars when compared with the uninoculated control. Expression levels were expressed as the Log_2_ (fold change) of DEGs. G9, *M. sativa* cv. Gannong No. 9; G3, *M. sativa* cv. Gannong No. 3; Q, *M. sativa* cv. Qingshui; L, *M. sativa* cv. Longzhong

Next, we compared the expression of genes with known functions in *Rhizobium*-legume symbiosis. As outlined in Fig. 7a, the nodule inception (NIN) gene (Medsa027206) was upregulated upon inoculation with six rhizobial strains. A total of 22 leghemoglobin genes were identified with 21 being upregulated in G9 (LL2 vs. CK, WLP2 vs. CK) and L (G3L3 vs. CK). Interestingly, gene Medsa009985 was upregulated in all treatments except G9 inoculated with the rhizobial strain LL2 (Fig. 7b). Surprisingly, all leghemoglobin genes (except Medsa009985) were downregulated and expressed at high levels in cultivars G3 (LL2 vs. CK, QL2 vs. CK) and Q (LL1 vs. CK). Cultivar-specific expression patterns were also observed for DEGs encoding glutamine synthetase (Fig. 7c).

### DEGs associated with flavonoid biosynthesis and plant-pathogen interaction

Given the established role of flavonoids in legume-rhizobial symbiosis, genes in the flavonoid biosynthesis pathway were further analyzed and results are summarized in Table 3. They were expressed in a strain-specific manner, including CHS (chalcone synthase), HCT (shikimate O-hydroxycinnamoyl transferase), DFR (dihydroflavonol 4-reductase), F3H (naringenin 3-dioxygenase) and E5.5.1.6 (chalcone isomerase). Expression levels of 41 DEGs differed significantly among the four alfalfa cultivars (Table 3). This suggests that rhizobial strains can potentially alter the patterns of flavonoid secretion during the process of symbiosis.

**Table 3:** The DEGs involved in flavonoid pathway in alfalfa cultivars upon inoculation of two rhizobial strains

Alfalfa cultivar	G9 ^**a**^	G3	Q	L	Gene	Gene product
Comparison	LL2 vs. WLP2	LL2 vs. QL2	WLP2 vs. LL1	G3L3 vs. LP3		
Medsa009599	−3.7 ^**b**^				CHS	Chalcone and stilbene synthase family protein
Medsa059715				−3.7	CHS	naringenin-chalcone synthase, partial
Medsa044389				−2.8	CHS	chalcone synthase 3, partial
Medsa086087				−2.5	CHS	naregenin-chalcone synthase, partial
Medsa009493				−2.1	CHS	Chain A, Chalcone Synthase--F215s Mutant
Medsa030411				−2	CHS	Chalcone synthase 2
Medsa026473				−1.9	CHS	chalcone and stilbene synthase family protein
Medsa044387				−1.7	CHS	chalcone synthase
Medsa026474				−1.6	CHS	chalcone synthase 4, partial
Medsa072385				− 1.4	CHS	chalcone synthase 3, partial
Medsa028257			4.4		F3H	naringenin 3-dioxygenase (flavanone-3-hydroxylase)
Medsa086086				−1.3	CHS	chalcone synthase
Medsa055925				−1.2	CHS	Chain A, Chalcone Synthase
Medsa055923				−2.2	CHS	chalcone synthase, partial
Medsa077779				−6.5	CHS	Chalcone synthase
Medsa063968				−1.5	CHS	chalcone-flavanone isomerase family protein
Medsa014983		−1.5			HCT	HXXXD-type acyl-transferase family protein
Medsa030991				1.7	HCT	hydroxycinnamoyl-CoA:shikimate hydroxycinnamoyl transferase
Medsa005120				1.4	HCT	hydroxycinnamoyl-CoA:shikimate hydroxycinnamoyl transferase
Medsa031435				−8.3	HCT	spermidine hydroxycinnamoyl transferase
Medsa024093				−2.8	DFR	dihydroflavonol 4-reductase
Medsa031435			−7.7		HCT	spermidine hydroxycinnamoyl transferase
Medsa016755				−6.3	HCT	HXXXD-type acyl-transferase family protein
Medsa018594		−1.9			DFR	dihydroflavonol 4-reductase-like protein
Medsa017082				−5.7	HCT	HXXXD-type acyl-transferase family protein
Medsa017084				−5.6	HCT	HXXXD-type acyl-transferase family protein
Medsa016758				− 5.4	HCT	HXXXD-type acyl-transferase family protein
Medsa032425				−5.1	HCT	anthranilate N-benzoyltransferase
Medsa017083				−5	HCT	HXXXD-type acyl-transferase family protein
Medsa084572				−4.3	HCT	anthranilate N-benzoyltransferase
Medsa016757				−4	HCT	anthranilate N-benzoyltransferase
Medsa084570				−2.9	HCT	anthranilate N-benzoyltransferase
Medsa046285				−1.9	HCT	HXXXD-type acyl-transferase family protein
Medsa076343				−3.4	HCT	HXXXD-type acyl-transferase family protein
Medsa020332			2.7		HCT	HXXXD-type acyl-transferase family protein
Medsa075370				−2.4	HCT	HXXXD-type acyl-transferase family protein
Medsa032175				−2	HCT	HXXXD-type acyl-transferase family protein
Medsa064961				−2.1	HCT	anthranilate N-benzoyltransferase
Medsa075250				−2.3	HCT	anthranilate N-benzoyltransferase
Medsa010989				1.6	HCT	anthranilate N-benzoyltransferase
Medsa019200				−6.6	E5.5.1.6	chalcone-flavanone isomerase family protein

^a^*M. sativa* cultivars, G9 for Gannong No. 9; G3 for Gannong No. 3; Q for Qingshui; L for Longzhong

^b^Data are Log_2_ (fold change) values for each differentially expressed gene (DEG)

To explore the potential of differential cell defense responses, plant-pathogen interaction pathways were further analyzed (Table 4). Data revealed a great variation in DEG numbers among the four cultivars. Only 10 DEGs were identified for G9, 53 for G3, and 64 for Q. However, a surprisingly high number of DEGs (722) identified in cultivar L belongs to this functional category. These DEGs were mostly downregulated relative to the uninoculated control. The most abundant DEGs in this category include RPM1 (resistance to *Pseudomonas syringae* pv. *maculicola* 1) and LRR-RLK (Leucine-rich repeat receptor-like kinase), which were commonly identified for all four cultivars. On the contrary, CERK1 (Chitin elicitor receptor kinase 1), CNGCs (Cyclic nucleotide gated channel), FLS2 (LRR receptor-like serine/threonine-protein kinase), HSP90B (Heat shock protein 90 kDa beta), LysM-RLK and PTI1 (Pto-interacting protein 1) were exclusively detected in only one cultivar. Together, these results suggest the potential roles of cellular defense mechanisms in the specific interaction below the species level.

**Table 4:** Number of differentially expressed genes involved in plant-pathogen interaction and those encoding plant peptide for an alfalfa cultivar upon inoculation of two rhizobial strains

Alfalfa cultivar		G9		G3		Q		L	
Rhizobial strains in comparison		LL2 vs. WLP2		LL2 vs. QL2		WLP2 vs. LL1		G3L3 vs. LP3	
DEG status		Up	Down	Up	Down	Up	Down	Up	Down
Genes involved in plant-pathogen interaction	CALM					3	1	2	28
	CDPK							1	16
	CERK1				1				
	CNGCs								6
	EDS1						1		1
	FLS2								6
	HSP90B					1			
	LRR-RLK		5	11	7	5	1	5	123
	LysM-RLK							1	6
	MEKK1				1				21
	PR1						3	1	
	PTI1								1
	RBOH			1					8
	RPP13	1			1				
	NBS-LRR		4	10	9	33	9	5	396
	NB-ARC			7	1	4	2	3	85
	WRKY25			1					6
	WRKY29			2	1				1
Plant peptide	CLE			1	1				
	GRPs						14		30
	NCRs	1	3				118		476
	PSK				1				
	RALF			1					9
	SNARPs						5		13

### DEGs encoding nodulins, peptides and transposons

A total of 35 nodulin-encoding DEGs were identified for cultivars G3, Q and L (Table 5), and none of them were shared between G3 (LL2 vs. QL2) and Q (WLP2 vs. LL1). Interestingly, DEGs identified in the G9 cultivar (LL2 vs. WLP2) don’t contain any genes involved in nodulin production. Thus, the data strongly suggest that nodulins are partially responsible for the specific below-species interactions. As shown in Table 4, a total of 673 peptide DEGs were identified, representing 8.3% of the total DEGs. Four types of peptides in 528 DEGs were found in L (G3L3 vs. LP3). The 137 DEGs identified for Q (WLP2 vs. LL1) belong to three types of peptide (NCR, GRP and SNARP). However, three distinct peptides were found for G3 (LL2 vs. QL2), i.e. CLE (CLAVATA3/ Embryo-Surrounding Region), PSK (Phytosulfokine) and RALF (Rapid Alkalinization Factor). Only NCR peptides were differentially regulated in G9 (LL2 vs. WLP2).

**Table 5:** Thirty-five differentially expressed nodulin genes identified in alfalfa cultivars upon inoculation of two rhizobial strains in comparison

Gene name	Gene code in alfalfa	G3 LL2 vs. QL2	Q WLP2 vs. LL1	L G3L3 vs. LP3
Early nodulin-12B	Medsa039711		−6.0	−8.7
Early nodulin-16	Medsa050076			−5.4
Early nodulin-20	Medsa041425		−9.7	−11.4
Early nodulin-75	Medsa030522	1.5 ^**a**^		
	Medsa041869	1.4		
Early nodulin-like protein	Medsa025394		−4.8	
Early nodulin-NMS-8	Medsa039710		−5.7	−10.7
Nodulin MtN21/EamA-like transporter family protein	Medsa034197	−3.1		
	Medsa063638	−2.0		
	Medsa036754	2.6		
	Medsa085504	2.3		
	Medsa051201	−1.7		
	Medsa039080	−1.7		
	Medsa050637		−4.9	−9.6
	Medsa042406		−3.1	
	Medsa078338		3.3	
	Medsa034378			−2.0
	Medsa020000		−9.5	−7.0
	Medsa074298			1.3
	Medsa037266			−8.8
	Medsa085797			1.3
Nodulin-1	Medsa050303			−7.1
Nodulin-22	Medsa004209			1.3
Nodulin-25	Medsa026012		−8.6	−13.0
	Medsa026013		−9.7	−13.2
	Medsa026014			−7.3
	Medsa062064		−11.7	−14.7
	Medsa062065			−11.2
Nodulin-26	Medsa026111		−3.4	−5.1
Nodulin-6	Medsa076070			−2.6
Nodulin-like/MFS transporter	Medsa081077			−2.1
	Medsa024741			−1.9
Vacuolar iron transporter-like protein	Medsa028561		−5.3	−10.2
	Medsa028596		−6.1	−9.5
	Medsa059717			−2.1

^a^ Log_2_ (fold change) value of differentially expressed genes. Nodulin genes were not found in DEGs of G9 (LL2 vs. WLP2)

Transposable elements are a universal feature of plant genomes, and alfalfa is not an exception [32]. Interestingly, a DEG encoding transposon (Medsa090988, Ty3-I Gag-polyprotein) was downregulated when cultivars G3 and Q were infected by an E1 and E2 biotype, i.e. LL2 vs. QL2 and WLP2 vs. LL1, respectively (Table 6). The distribution of DEGs encoding 15 proteins commonly identified in all four cultivars is provided in Additional file 13.

**Table 6:** Twenty-one genes associated with the cultivar-specificity spectrum of biotype strains

Gene	Description	G3 (LL2 vs. QL2)	Q (WLP2 vs. LL1)
Medsa042668	ubiquitin1	5.8 ^**a**^	3.5
Medsa090988	Transposon Ty3-I Gag-Pol polyprotein, partial	−4.5	−2.0
Medsa070600	subtilisin-like serine protease	−1.5	−1.1
Medsa033982	Sporozoite surface protein 2	−1.8	−1.5
Medsa017908	transcription factor bHLH100	−2.0	−2.0
Medsa054952	NADP-dependent glyceraldehyde-3-phosphate dehydrogenase	1.8	2.7
Medsa047698	Kunitz type trypsin inhibitor / Alpha-fucosidase	2.9	2.4
Medsa045026	hypothetical protein TSUD_339160	2.5	2.7
Medsa031174	hypothetical protein MTR_3g111095	−5.1	−5.8
Medsa033325	horseradish peroxidase-like protein	2.1	2.4
Medsa009230	DUF1442 family protein	−1.3	−1.6
Medsa041850	disease resistance protein (TIR-NBS-LRR class)	1.7	2.1
Medsa041941	disease resistance protein (TIR-NBS-LRR class)	3.5	4.0
Medsa088244	disease resistance protein (TIR-NBS-LRR class)	7.0	8.0
Medsa057523	disease resistance protein (TIR-NBS-LRR class)	4.6	2.5
Medsa024638	cytochrome P450 family ent-kaurenoic acid oxidase	2.6	4.0
Medsa003249	branched-chain amino acid aminotransferase	3.3	4.3
Medsa035785	alpha-amylase carboxy-terminal beta-sheet domain protein	−4.5	−6.7
Medsa017564	Unknown	7.5	3.9
Medsa033887	Unknown	−4.8	−3.7
Medsa049192	Unknown	9.2	12.1

^a^ Log_2_ (fold change) is the average value retrieved by RNA-seq analysis of three biological replicates of the *M. sativa* cvs. Gannong No. 3 (G3) and Qingshui (Q) upon inoculation of effective two-cultivars specific biotype (E2) and effective one-cultivar specific biotype (E1) strains

### Validation of DEGs by *q*RT-PCR

Primers were designed to verify nine DEGs using *q*RT-PCR (Additional file 14). Seven genes (Medsa004474, Medsa025205, Medsa053760, Medsa057934, Medsa062433, Medsa067873 and Medsa084795) were commonly expressed in G3 (LL2 vs. QL2), Q (WLP2 vs. LL1), and L (G3L3 vs. LP3), whereas Medsa002736 was identified in G9 (LL2 vs. WLP2), Q (WLP2 vs. LL1) and L (G3L3 vs. LP3). Medsa002106 was commonly expressed in G9 (LL2 vs. WLP2), G3 (LL2 vs. QL2) and Q (WLP2 vs. LL1). Results of *q*RT-PCR were in good accordance with expectations from the RNA-seq data (Additional file 15).

## Discussion

The present study sought to extend our understanding of the specific *Rhizobium*-legume interactions to the below-species levels. To this end, we first identified 12 *E. melilti* biotypes on the basis of their symbiotic performance on five alfalfa cultivars. Biotype classification is an in-depth identification of intraspecies rhizobia at the intraspecies level of the host plants [28, 33]. Next, we used the most advanced RNA-seq technique to dissect the plant transcriptomic responses to rhizobial infections. The experiments were designed to test whether a single plant cultivar significantly displays different patterns of gene expression upon infections by two closely related rhizobial strains. First of all, our results of RNA-seq revealed significant difference of plant transcriptomes for the four alfalfa cultivars each inoculated with two rhizobial strains in comparison. The number of DEGs reached up to 12.2% of the entire set of genes encoded in the genome of *M. sativa*. More importantly, the number of DEGs and functional pathways were positively correlated with the phylogenetic distance between the two rhizobial strains. Our work also identified candidate genes associated with specific intraspecies interactions. Such information lay the foundation for further analysis to understand the molecular mechanisms underlying the intraspecies specificity. Moreover, the biotype affiliation has direct implications for practical use of biological nitrogen fixation in agriculture.

### Biotype identification on the basis of symbiotic performance

The symbiotic efficiency is an estimate of host growth promotion and is normally associated with enhanced plant SDW [34, 35]. Here, we show that the SDW-based symbiotic efficiency of *E. meliloti* strains varied greatly across the five alfalfa cultivars. The data strongly implicate a host-driven adaptative evolution of rhizobial populations in soil. Interestingly, some strains showed inhibitory effects on certain plant cultivars. The potentially beneficial bacteria can thus act in a parasitic (or pathogenic) manner [36, 37]. Under certain conditions carbon rather than nitrogen can be a limiting factor for plant growth. The observed noneffective (O), inhibitory (I) and effective (E) intraspecies interactions can thus be explained by the equilibrium between the energy cost of N_2_-fixation and mineral nitrogen acquisition [38]. Moreover, our results of SDW-based bio-typing are generally consistent with previous reports in regards to the use of plant dry matter biomass for evaluating symbiotic efficiency [35, 39, 40]. It is thus critically important to inoculate compatible rhizobial strains when a specific alfalfa cultivar is cultivated in agriculture.

Another interesting finding from this study is that some rhizobial biotypes formed efficient symbiosis with newly introduced alfalfa cultivars, but not with plant cultivar from which they were initially isolated. While this may be caused by the differences between laboratory plant growth conditions and the natural environments, highly efficient strain-specific legume-rhizobial interactions can occur without a long-term coevolution in the same habitats [7]. Thus, a large collection of diverse rhizobial strains will help select for compatible biotypes for specific alfalfa cultivars [28].

### Variation of plant transcriptomic responses to rhizobial infections at the below-species level

This work involved five alfalfa cultivars belonging to the same species of *M. sativa*. Cultivar WL is phylogenetically distinct from the four cultivars used for RNA-seq (Fig. 3), and it didn’t form effective symbiosis with any of the 32 *E. meliloti* strains. This indicates that WL, as a newly introduced alfalfa cultivar in this area, has weak adaptability, compatibility and kinship relationship with native rhizobial resources. Conversely, domestic hybrid cultivars G3 and G9 as well as local cultivars L and Q appear to have well adapted to the local environments as they are capable of forming specific and effective symbiosis with native rhizobial strains. Cultivar WL was excluded from the present transcriptome analysis, but the mechanisms underlying the observed inhibitory/noneffective symbiosis of WL with all local rhizobial isolates warrant further investigation in separate studies.

With regards to the specific DEGs, our results are generally consistent with previous reports on strain specific symbiotic interactions between *Azospirillum* and rice and also between *Rhizobium* and soybean [25, 41]. Most DEGs are associated with starch and sucrose metabolism, ribosome, plant hormone signal transduction, plant-pathogen interaction and biosynthesis of flavonoids and other secondary metabolites [25, 41]. In this study, a relatively small number of DEGs (0.5%) was detected for G9 cultivar between the two E2 rhizobial strains (LL2 vs. WLP2), but they contain more DEGs for leghemoglobin and glutamine synthetase when compared with G3 and Q cultivars: G3 (LL2 vs. CK) and Q (WLP2 vs. CK). Leghemoglobin and glutamine synthetase play key roles in modulating oxygen availability and nitrogen metabolism, respectively [42, 43]. Significantly, all leghemoglobin genes detected in G9 were upregulated, but those detected in G3 were downregulated except Medsa009985. This may explain the finding that E2-induced G9 nodules had much higher shoot dry weight and stronger nitrogen fixing abilities than E1-induced G3 nodules [44].

In accordance with our previous conclusion, alfalfa cultivars differ in their sensitivity to infections by different rhizobial strains [45, 46]. However, it remains unclear if the extent of the transcriptomic variation is correlated with the phylogenetic distance among the rhizobial symbionts. In this study, we compared the transcriptomes of one plant cultivar (L) inoculated with two rhizobial strains belonging to the same biotype (G3L3 vs. LP3 in biotype III). A surprisingly high number of DEGs (5816 in total) were detected, representing 12.2% of the *M. truncatula* genome. Twenty-two leghemoglobin genes were upregulated upon infections by G3L3 relative to LP3. Other DEGs includes those associated with plant immunity and metabolism of substrates that are crucial for nodulation and N-fixation, such as polysaccharide, phosphorus, calcium, ion, carbohydrate and sulfate. Together, the data provide empirical evidence to support the hypothesis of strain-specific molecular interactions in *Rhizobium*-legume symbiosis.

### Potential role of plant innate immunity in determining the specificity of *Rhizobium*-legume symbiosis

Plant innate immunity is triggered at the initial stage of rhizobial infection, but rhizobial cells can actively suppress or evade the plant innate immune system to avoid being targeted as invading pathogens by their compatible hosts [47]. Previous studies showed that LRR-RLKs were accumulated in nodules formed by the *symCRK* mutant of *M. truncatula* [48]. The soybean NBS-LRR resistance (*R*) genes determine host-rhizobia specificity through the recognition of effector proteins delivered by the rhizobial type III secretion systems [49]. The *R* genes are potentially responsible for the exclusion of ineffective rhizobial strains by alfalfa [50, 51]. However, in mature nodules defense reactions can potentially kill bacteria and block the trophic exchanges between bacteroids and their legume host [14, 52]. In this study, several defense related genes were detected, and they were primarily downregulated in effective nodules of the G9 and L cultivars (Table 4). These include LRR-RLK, NBS-LRR and NB-ARC, which are major *R*-genes for effector triggered immunity [41, 53]. This finding strongly implicates the roles of plant innate immunity in the specific perception of rhizobial strains.

## Conclusion

Data presented here reveal specific symbiotic interactions between alfalfa cultivars and *E. meliloti* strains that were classified into 12 symbiotic biotypes. More significantly, we show that alfalfa cultivar displays distinct transcriptomic profiles in response to infections by rhizobial isolates at the below-species levels (i.e. biotype, strain). Differentially expressed genes include Medsa002106 and those encoding nodulins and NCR peptides and NBS-LRR proteins. Further analysis of the identified DEGs will provide deeper insights into the underlying mechanisms of the below-species symbiotic specificities.

## Methods

### Plant sampling and genetic identification

Alfalfa seeds and plants with rhizosphere soil were collected in May and August 2014 from three pasture experimental stations administrated by Gansu Agricultural University, Gansu, China (Additional file 1) using standard methods as previously described [45, 46]. For phylogenetic analysis, genomic DNAs were extracted from leaf samples and subsequently used for PCR amplification of four housekeeping genes *matK1*, *matK2*, *matK3*, and *rbcL* as previously described [54]. DNA sequencing was performed using the services of TSINGKE Biological Technology Company (Xian, Shan’xi, China). Neighbor-Joining trees were constructed using the MEGA 6.0 software [46, 55]. Voucher specimens for the five alfalfa cultivars are deposited in the cognitive pavilion herbarium of Gansu Agricultural University with accession numbers from ms-20140421-01 to ms-20140421-05 for plants, and ms-20140813-04 to ms-20140813-08 for seeds.

### Symbiotic performance analysis of *E. meliloti* isolates

Rhizobia were isolated from various types of samples such as nodule, leaf, stem, flower, root epidermis, root stele, rhizosphere soil, field soil and seed [45]. All the 32 isolates were subjected to identification by 16S rRNA gene sequencing and subsequent phylogenetic analysis using the standard methods [46].

The symbiotic performance was determined on five alfalfa cultivars using a completely randomized design model [56]. Briefly, seeds were surface-sterilized by immersion in iodophor disinfectant (containing 2500 mg·mL^− 1^ available iodine) for 2 min, then rinsed with sterile distilled water five times and dried thoroughly. Next, seeds were germinated on 0.8% (m·v^− 1^) water agar at 28 °C for 24 h, and then transplanted into a plastic pot (diameter: 13.2 cm, height: 10 cm) at a depth of 2 cm. Each pot contained 450 g of sterilized sand. The sand was screened with a 2-mm sieve, soaked in 1 mol·L^− 1^ HCl to reach pH 7, rinsed seven times with distilled water, dried in an oven at 105 °C, and sterilized in an autoclave at 121 °C for 6 h [56]. Pots were placed in a plastic basin (29 cm × 20 cm × 9.5 cm) with 500 mL of sterile distilled water and irrigated with 500 mL of Hoagland nitrogen solution on the seventh day [57]. The basins were then placed in a growth chamber with a day: night cycle of 16 h: 8 h, with temperatures of 22 °C during the day and 18.5 °C at night. Relative humidity was set at 45 ± 5% and the light was 150 μmol·m ^− 2^·s ^− 1^.

Plants were inoculated 2 weeks after germination with the emergence of first leaf for > 90% seedlings. Inoculants were prepared by first growing *E. meliloti* strains preserved in − 80 °C freezer in 50 mL TY broth medium [58] at 28 °C on a rotary shaker (180 rpm) for 24 ~ 48 h to reach a full growth (OD_600nm_ = 1). Each culture was then centrifuged at 10000 rpm, 25 °C for 10 min (Centrifuge Xiangyi, H1650, Changsha, China) and re-suspended in sterilized distilled water to an OD_600nm_ of 0.5 [56]. All alfalfa seedlings (~ 30) in one pot were inoculated at the same time using 30 mL of rhizobial suspension. Four independent biological replicates (pots) were set up for each treatment. After inoculation, pots were irrigated with 500 mL of Hoagland N-free nutrient solution once a week. The daily consumption of water in the basin was supplemented with sterile distilled water. At 45 days after inoculation ten seedlings were randomly selected from each pot and symbiotic properties were assayed using standard methods [45]: nodule number, effective nodule weight (the weight of pink nodules), nodule diameter, compound leaf number, shoot height, root length, nodule nitrogenase activity, chlorophyll content and crude protein content. Fresh and dry weights were measured separately for the shoot and root of a plant. Nodules were graded as previously described [59]. Briefly, the wizened death nodules were defined as grade 1; nodules with white transverse section were defined as grade 2; pink nodules with diameter < 0.5 mm were defined as grade 3; pink nodules whose diameter were 0.5 ~ 1 mm were defined as grade 4; pink nodules with diameter > 1 mm were defined as grade 5. Symbiotic efficiency of strains was marked as effective (E), noneffective (O), or inhibitory (I), which represent significantly higher, none or lower symbiotic values (*P* < 0.05) when compared with the uninoculated plants.

### RNA-seq analysis

As outlined in Fig. 3, RNA-seq was performed with 12 treatments with three independent replicates. It involved four alfalfa cultivars, and each was inoculated with two rhizobial strains plus an uninoculated control. All rhizobial strains can form effective nodules on the related cultivars. Roots with nodules were harvested at 45 days after inoculation and immediately frozen in liquid nitrogen. Total RNAs were extracted using the EASYspin Plus Plant RNA Isolation kit (Aidlab, Beijing, China) according to the manufacturer’s protocol. The RNA concentration was spectrophotometrically quantified (NanoDrop Technologies, Inc.), and its integrity was determined using an Agilent 2100 Bioanalyzer (Agilent Technologies, Inc.).

Libraries were generated using the NEBNext® Ultra™ RNA Library Prep Kit for Illumina® (NEB, Ipswich, MA, USA) following the manufacturer’s instructions. Briefly, mRNA was enriched with oligo (dT)-attached magnetic beads, and the first strand cDNA was synthesized using six bases of random hexamer primers and M-MuLV reverse transcriptase. The second strand cDNA synthesis was subsequently performed using buffer solution, dNTPs, RNase H and DNA polymerase I. After purification by a QiaQuick PCR kit (Borunlaite, Beijing, China) and elution by EB buffer solution, the remaining cDNA fragment overhangs were repaired to blunt ends. The 3′ ends of DNA fragments were adenylated before sequence adaptor ligation with a hairpin loop structure. Agarose gel electrophoresis was then conducted to select for cDNA fragments of 150 ~ 200 bp in length. Finally, PCR was performed with Phusion High-Fidelity DNA polymerase, universal PCR primers and index (X) primers. A total of 36 cDNA libraries (12 treatments × 3 replicates) were sequenced at both ends on the Illumina NovaSeq 6000 platform using the serves provided by Sagene Biotech Co., Ltd. (Guangzhou, China).

### Determination of differentially expressed genes

Raw RNA-seq data in fastq format were first processed using FastQC (v0.11.5) to remove the adapters and sequences of low quality. Due to the absence of reference genomic sequences, high-quality clean reads were assembled using Trinity (v2.2.0) software as described previously [60]. Sequences with similarity > 95% were grouped into one class, and the longest sequence of each class was treated as the unigene in subsequent processing. Taxonomic and functional annotation of the transcripts were performed using Blast+ (v2.4.0) for the annotation with Nr (non-redundant protein sequences from NCBI), Swiss-Prot (a manually annotated and non-redundant protein sequence database) and COG/KOG (cluster of orthologous groups of proteins), KAAS for annotation with KEGG, Blast2GO (v2.3.5) for GO annotation and HMMER3 for Pfam (protein families database of alignments and hidden Markov models). Genes were identified with an E-value 10^− 5^ against sequences deposited in the database.

Full-length reads were directly mapped to the reference unigenes using the RSEM software package v1.2.31 [61]. Genes with FDR < 0.05 and log_2_(FC) ≥ 1 were designated DEGs [62], which were determined using EdgeR v3.14.0 [63]. GO enrichment analysis of all DEGs was implemented by using Blast2GO (v2.3.5), and KEGG pathway enrichment analysis of the DEGs was performed using KEGG (http://www.expasy.org). A hypergeometric test was used to identify the significantly enriched GO functions and KEGG pathways. The calculated *p*-value was subjected to Bonferroni correction, taking a corrected *p*-value < 0.05 as a threshold. The *q*-value is defined as a natural pFDR (positive FDR) analogue to the *p*-value [64], and a significant level of 0.05 was selected for the enrichment analysis.

### Quantitative real-time PCR (*q*RT-PCR)

Total RNAs were prepared using the method described above and *q*RT-PCR was performed using standard procedures with β-actin as an endogenous control. Sequences of the oligonucleotide primers are provided in Additional file 14. The *q*RT-PCR data were analysed by melting curve analysis based on the △△CT and 2^-△△CT^ methods [65]. The △CT value of each gene was calculated by subtracting the CT value of the endogenous control from the CT value of the target gene. Statistical analysis was performed in Prism version 8.0 (GraphPad Software Inc., San Diego, California, USA).

## Supplementary information

**Additional file 1. **List of the *Ensifer meliloti* strains.

**Additional file 2. **A representative phylogenetic tree for 32 *Ensifer meliloti* strains based on sequences of the 16S rRNA genes. Accession numbers in GenBank (www.ncbi.nlm.nih.gov/) are shown in brackets.

**Additional file 3. **Symbiotic performance of 32 *Ensifer meliloti* strains on *Medicago sativa* cv. Gannong No. 9. *E. meliloti* strains are coded from 1 to 32 in the following order: G3L2, G3L3, G3L4, G3L5, G3L6, G3L7, G3L8, G3L9, G3L10, G3L12, G3L13, G3T2, G9L3, G9L4, G9L5, G9L6, G9L7, G9L8, LL1, LL2, LL5, LL6, LL7, LL8, LL10, LL11, LP3, QL2, QL4, QL5, WLG1 and WLP2. Red and green boxes indicate significant higher and lower values than the uninoculated control, respectively (*P* < 0.05). Strains subjected to transcriptome analysis are marked with blue lines and purple boxes (****, significance at *P* < 0.0001; ns, no significance at *P* < 0.05). Data are means and standard errors of four biological replicates.

**Additional file 4. **Symbiotic performance of 32 *Ensifer meliloti* strains on *Medicago sativa* cv. Gannong No. 3. *E. meliloti* strains are coded from 1 to 32 in the following order: G3L2, G3L3, G3L4, G3L5, G3L6, G3L7, G3L8, G3L9, G3L10, G3L12, G3L13, G3T2, G9L3, G9L4, G9L5, G9L6, G9L7, G9L8, LL1, LL2, LL5, LL6, LL7, LL8, LL10, LL11, LP3, QL2, QL4, QL5, WLG1 and WLP2. Red and green boxes indicate significant higher and lower values than the uninoculated control, respectively (*P* < 0.05). Strains subjected to transcriptome analysis are marked with blue lines and purple boxes (****, significance at *P* < 0.0001; ns, no significance at *P* < 0.05). Data are means and standard errors of four biological replicates.

**Additional file 5. **Symbiotic performance of 32 *Ensifer meliloti* strains on *Medicago sativa* cv. Qingshui. *E. meliloti* strains are coded from 1 to 32 in the following order: G3L2, G3L3, G3L4, G3L5, G3L6, G3L7, G3L8, G3L9, G3L10, G3L12, G3L13, G3T2, G9L3, G9L4, G9L5, G9L6, G9L7, G9L8, LL1, LL2, LL5, LL6, LL7, LL8, LL10, LL11, LP3, QL2, QL4, QL5, WLG1 and WLP2. Red and green boxes indicate significant higher and lower values than the uninoculated control, respectively (*P* < 0.05). Strains subjected to transcriptome analysis are marked with blue lines and purple boxes (****, significance at *P* < 0.0001; ns, no significance at *P* < 0.05). Data are means and standard errors of four biological replicates.

**Additional file 6. **Symbiotic performance of 32 *Ensifer meliloti* strains on *Medicago sativa* cv. Longzhong. *E. meliloti* strains are coded from 1 to 32 in the following order: G3L2, G3L3, G3L4, G3L5, G3L6, G3L7, G3L8, G3L9, G3L10, G3L12, G3L13, G3T2, G9L3, G9L4, G9L5, G9L6, G9L7, G9L8, LL1, LL2, LL5, LL6, LL7, LL8, LL10, LL11, LP3, QL2, QL4, QL5, WLG1 and WLP2. Red and green boxes indicate significant higher and lower values than the uninoculated control, respectively (*P* < 0.05). Strains subjected to transcriptome analysis are marked with blue lines and purple boxes (****, significance at *P* < 0.0001; ns, no significance at *P* < 0.05). Data are means and standard errors of four biological replicates.

**Additional file 7. **Symbiotic performance of 32 *Ensifer meliloti* strains on *Medicago sativa* cv. WL168HQ. *E. meliloti* strains are coded from 1 to 32 in the following order: G3L2, G3L3, G3L4, G3L5, G3L6, G3L7, G3L8, G3L9, G3L10, G3L12, G3L13, G3T2, G9L3, G9L4, G9L5, G9L6, G9L7, G9L8, LL1, LL2, LL5, LL6, LL7, LL8, LL10, LL11, LP3, QL2, QL4, QL5, WLG1 and WLP2. Red and green boxes indicate significant higher and lower values than the uninoculated control, respectively (*P* < 0.05). Strains subjected to transcriptome analysis are marked with blue lines and purple boxes (****, significance at *P* < 0.0001; ns, no significance at *P* < 0.05). Data are means and standard errors of four biological replicates.

**Additional file 8. **Assessment of the symbiotic efficiency for 32 *Ensifer meliloti* strains on five alfalfa cultivars. Strains were placed into the following three categories: effective (E), noneffective (O) and inhibitory (I) with shoot dry weight values significantly higher, no significant difference and significantly lower than that of the uninoculated control plants, respectively (*P* < 0.05).

**Additional file 9. **Summary of assembled transcriptome and function annotation of *Medicago sativa*.

**Additional file 10. **Length distribution of de novo assembly and taxonomic source of BLAST matches for *Medicago sativa* unigenes. **a** Length distribution of de novo assembly; **b** Taxonomic source of BLAST matches.

**Additional file 11. **Gene ontology (GO) distributions and KEGG classification of the *Medicago sativa* transcriptomes. a The main functional categories in the biological process, cellular component and molecular functions categories found in the transcriptome. The ordinate indicates the number of unigenes. Bars represent the numbers of assignments of *M. sativa* proteins with BLASTx matches to each GO term. One unigene may be matched to multiple GO terms. b The left Y-axis indicates the KEGG pathway. Unigenes involved in metabolism pathways by KEGG classification are divided into five groups as shown on the right Y-axis. The X-axis indicates the percentage of unigenes that were assigned to a specific pathway.

**Additional file 12.** Common genes expressed for an alfalfa cultivar inoculated with two rhizobial strains.

**Additional file 13.** Numbers of up- and down-regulated DEGs commonly identified in four cultivars. Numbers of up- and down-regulated DEGs are shown for 15 proteins, except plant peptides and those involved in plant-pathogen interactions.

**Additional file 14. **Sequence of primers used in this work for quantitative real-time PCR (*q*RT-PCR).

**Additional file 15. **Validation of nine DEGs by Quantitative real-time PCR (*q*RT-PCR). The *q*RT-PCR data are means ± SEM of log_2_ (fold change) calculated using the 2^-△△CT^ method. **a-g** seven genes jointly expressed in G3, Q and L. **h** one gene jointly expressed in G9, Q and L. **i** one gene jointly expressed in G9, G3 and L.

## Data Availability

The RNA-seq data have been deposited in GenBank with accession numbers SRR8224042 to SRR8224077. Sequences of the four house-keeping genes are available in GenBank repository under accession numbers MN159019 to MN159037.

## Abbreviations

NCRs: Nodule-specific cysteine-rich peptides
GRPs: Nodule-specific glycine-rich proteins
SNARPs: Small nodulin acidic RNA binding proteins
NFs: Nod factors
SDW: Shoot dry weight
E1: Effective one-cultivar specific biotype
E2: Effective two-cultivar specific biotype
NBS-LRR: Nucleotide-binding domain leucine rich repeat
