# Supplementary material for: Plant transcriptome analysis reveals specific molecular interactions between alfalfa and its rhizobial symbionts below the species level
Source: BMC Plant Biol. 2020 Jun 26;20:293. doi: 10.1186/s12870-020-02503-3 (PMC7318466; doi:10.1186/s12870-020-02503-3)
Supplement: Supplementary file 1 — Additional file 1. List of the Ensifer meliloti strains. [file 12870_2020_2503_MOESM1_ESM.pdf]

Additional file 1: List of the *Ensifer meliloti* strains

Isolate [a]	Isolation spots	*Medicago sativa* cultivar	Site	Geographical position	Soil texture	Annual rainfall (mm)
G3L2, G3L3, G3L4, G3L5, G3L6, G3L7, G3L8, G3L9, G3L10, G3L12, G3L13	Nodule	Gannong No. 3 (G3)	Wuwei, Gansu, China	E 102°50′, N 37°52′, 1650 m	Gray brown desert	158
G3T2	Rhizosphere soil					
G9L3, G9L4, G9L5, G9L6, G9L7, G9L8	Nodule	Gannong No. 9 (G9)				
LL1, LL2, LL5, LL6, LL7, LL8, LL10, LL11	Nodule	Longzhong (L)	Huining, Gansu, China	E 105°06′, N 34°40′, 1760 m	Loess sandy loam	300
LP3	Epidermis					
QL2, QL4, QL5	Nodule	Qingshui (Q)				
WLG1	Stele	WL168HQ (WL)	Lanzhou, Gansu, China	E 105°41′, N 34°05′, 1517 m	Loess loam	320
WLP2	Epidermis					

a A prefix was assigned to inform plant cultivar from which the strains was originally isolated: G3, Gannong No. 3; G9, Gannong No. 9; L, Longzhong; Q, Qingshui; WL, WL168HQ.
