# Supplementary figures and images for: Plant transcriptome analysis reveals specific molecular interactions between alfalfa and its rhizobial symbionts below the species level
Source: BMC Plant Biol. 2020 Jun 26;20:293. doi: 10.1186/s12870-020-02503-3 (PMC7318466; doi:10.1186/s12870-020-02503-3)

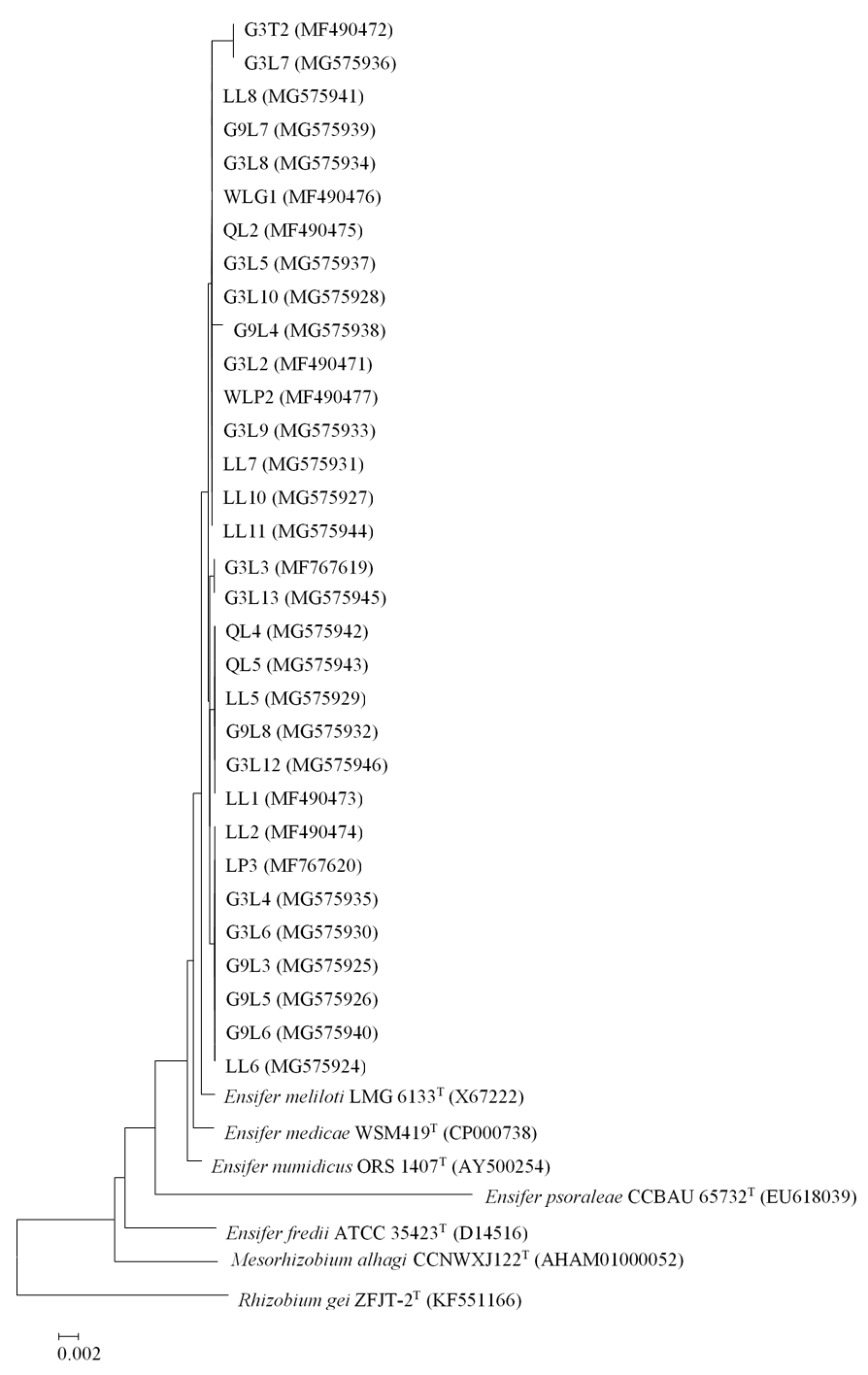

Supplement: Supplementary file 2 — Additional file 2. A representative phylogenetic tree for 32 Ensifer meliloti strains based on sequences of the 16S rRNA genes. Accession numbers in GenBank (www.ncbi.nlm.nih.gov/) are shown in brackets. [file 12870_2020_2503_MOESM2_ESM.tif]

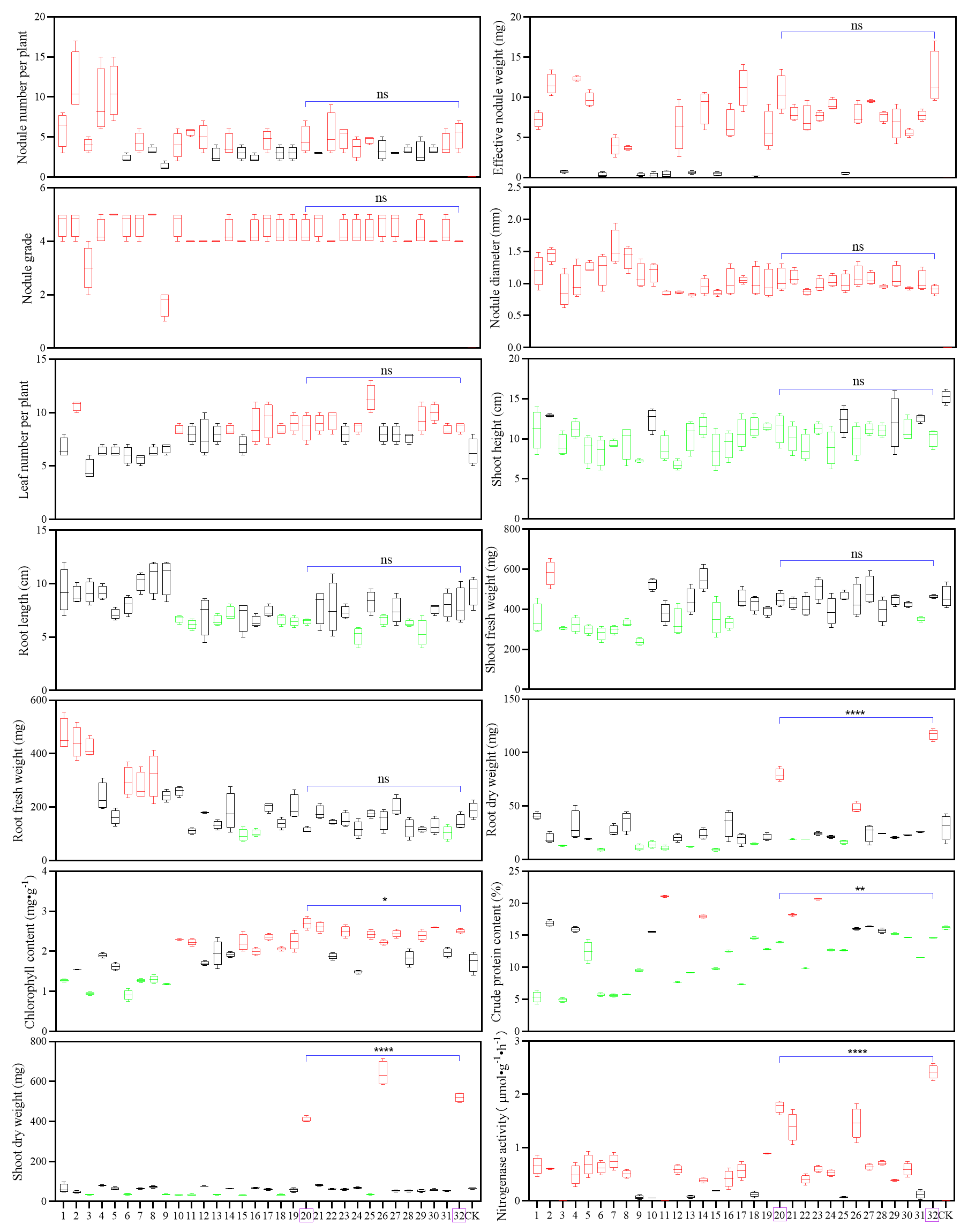

Supplement: Supplementary file 3 — Additional file 3. Symbiotic performance of 32 Ensifer meliloti strains on Medicago sativa cv. Gannong No. 9. E. meliloti strains are coded from 1 to 32 in the following order: G3L2, G3L3, G3L4, G3L5, G3L6, G3L7, G3L8, G3L9, G3L10, G3L12, G3L13, G3T2, G9L3, G9L4, G9L5, G9L6, G9L7, G9L8, LL1, LL2, LL5, LL6, LL7, LL8, LL10, LL11, LP3, QL2, QL4, QL5, WLG1 and WLP2. Red and green boxes indicate significant higher and lower values than the uninoculated control, respectively (P < 0.05). Strains subjected to transcriptome analysis are marked with blue lines and purple boxes (****, significance at P < 0.0001; ns, no significance at P < 0.05). Data are means and standard errors of four biological replicates. [file 12870_2020_2503_MOESM3_ESM.tif]

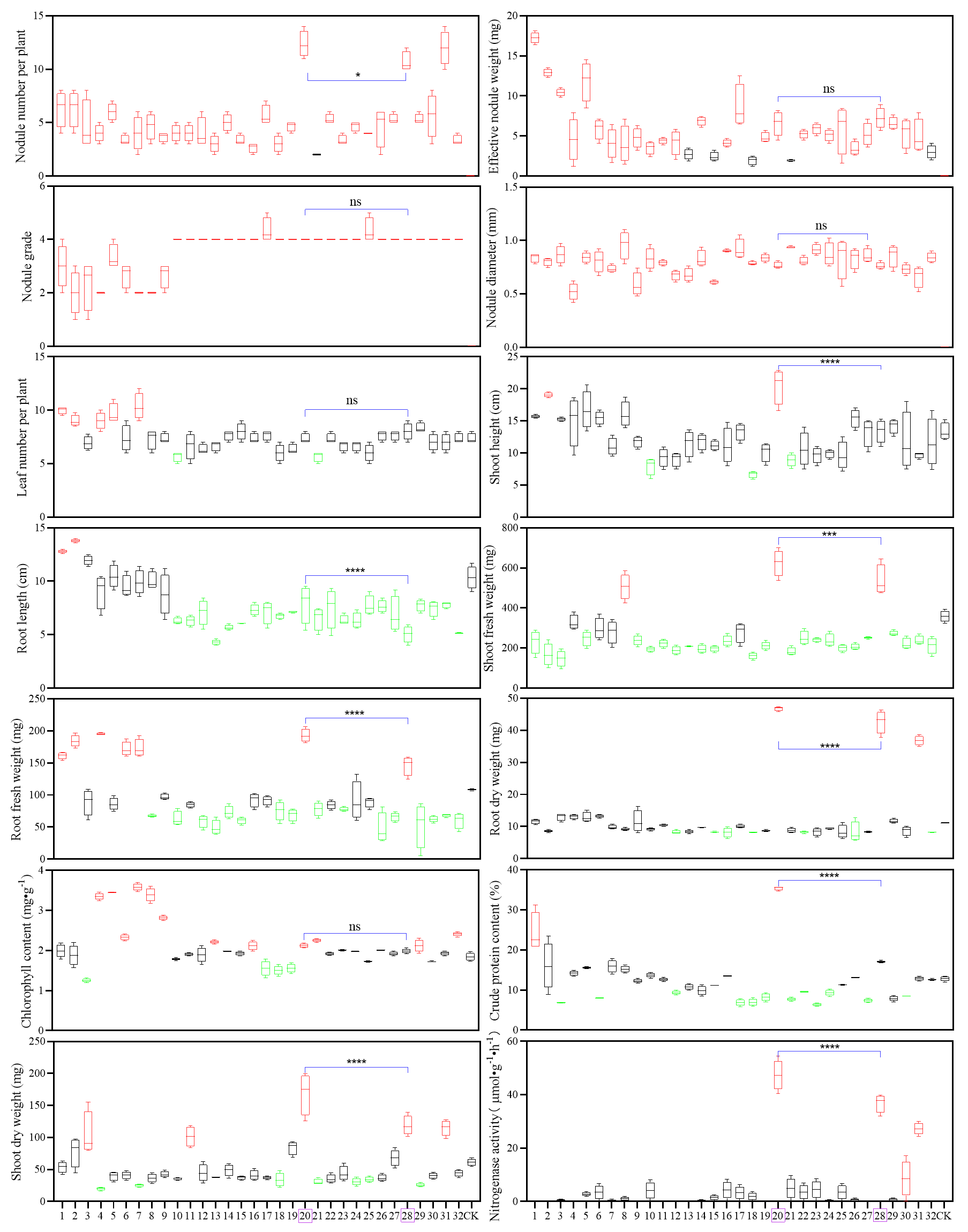

Supplement: Supplementary file 4 — Additional file 4. Symbiotic performance of 32 Ensifer meliloti strains on Medicago sativa cv. Gannong No. 3. E. meliloti strains are coded from 1 to 32 in the following order: G3L2, G3L3, G3L4, G3L5, G3L6, G3L7, G3L8, G3L9, G3L10, G3L12, G3L13, G3T2, G9L3, G9L4, G9L5, G9L6, G9L7, G9L8, LL1, LL2, LL5, LL6, LL7, LL8, LL10, LL11, LP3, QL2, QL4, QL5, WLG1 and WLP2. Red and green boxes indicate significant higher and lower values than the uninoculated control, respectively (P < 0.05). Strains subjected to transcriptome analysis are marked with blue lines and purple boxes (****, significance at P < 0.0001; ns, no significance at P < 0.05). Data are means and standard errors of four biological replicates. [file 12870_2020_2503_MOESM4_ESM.tif]

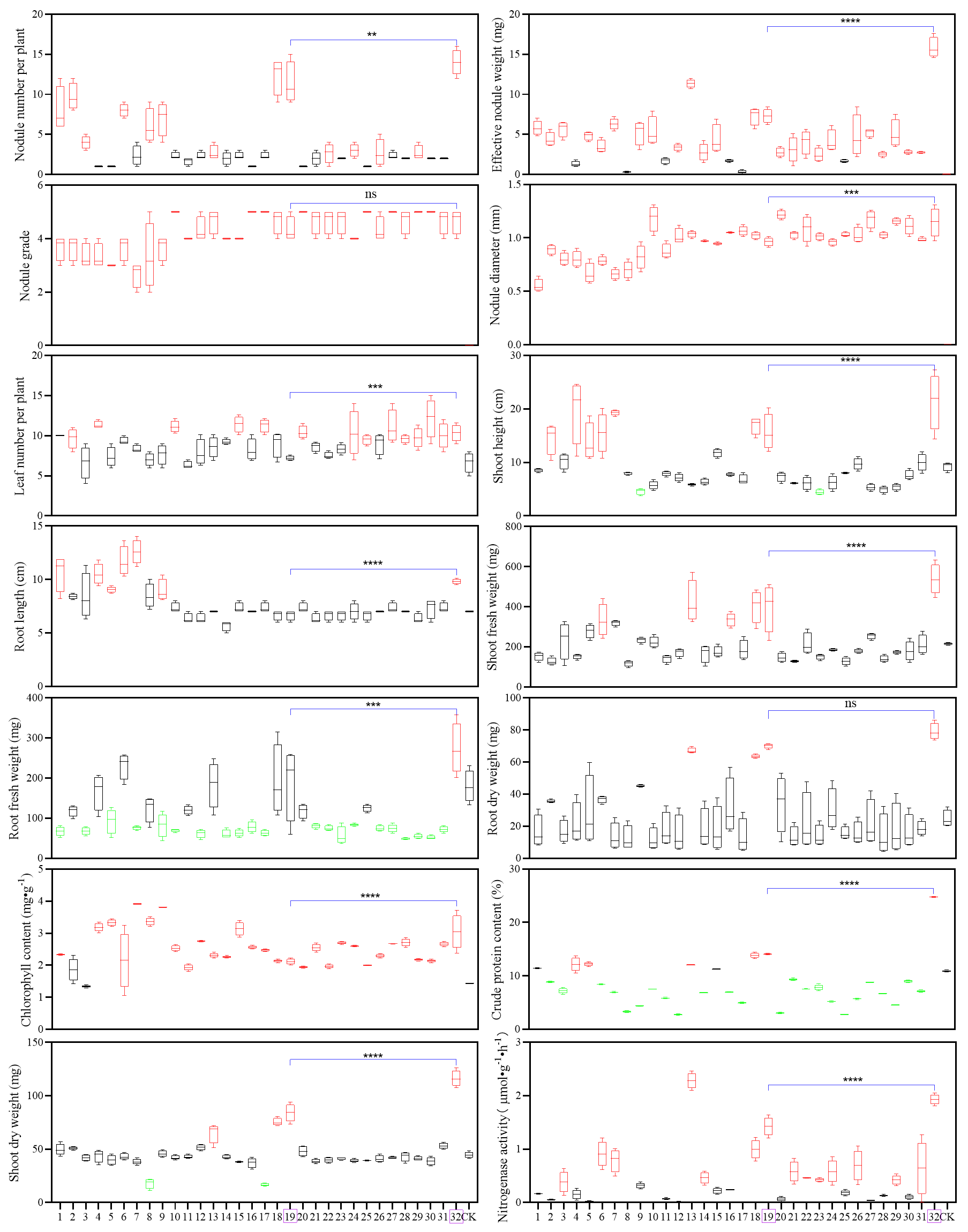

Supplement: Supplementary file 5 — Additional file 5. Symbiotic performance of 32 Ensifer meliloti strains on Medicago sativa cv. Qingshui. E. meliloti strains are coded from 1 to 32 in the following order: G3L2, G3L3, G3L4, G3L5, G3L6, G3L7, G3L8, G3L9, G3L10, G3L12, G3L13, G3T2, G9L3, G9L4, G9L5, G9L6, G9L7, G9L8, LL1, LL2, LL5, LL6, LL7, LL8, LL10, LL11, LP3, QL2, QL4, QL5, WLG1 and WLP2. Red and green boxes indicate significant higher and lower values than the uninoculated control, respectively (P < 0.05). Strains subjected to transcriptome analysis are marked with blue lines and purple boxes (****, significance at P < 0.0001; ns, no significance at P < 0.05). Data are means and standard errors of four biological replicates. [file 12870_2020_2503_MOESM5_ESM.tif]

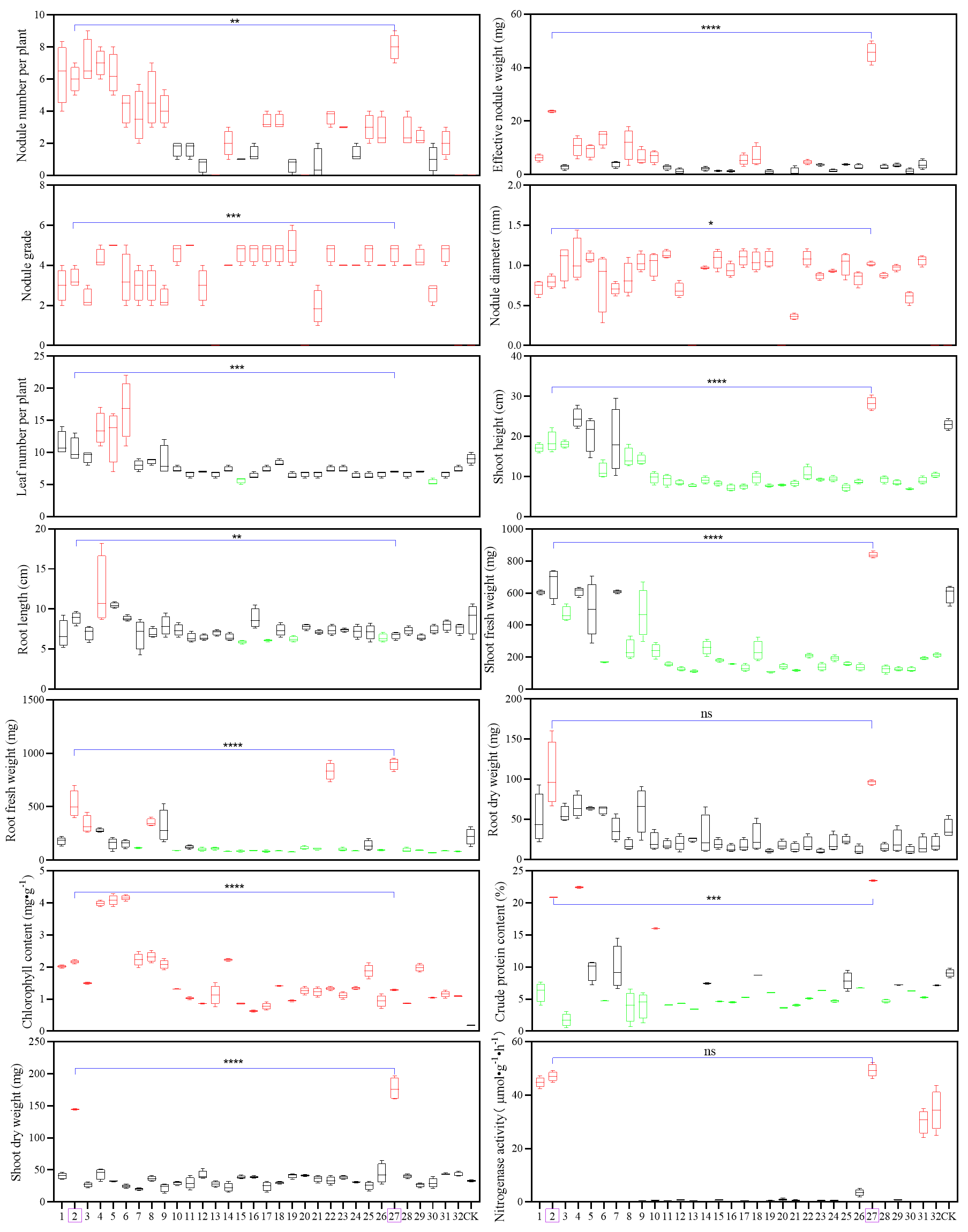

Supplement: Supplementary file 6 — Additional file 6. Symbiotic performance of 32 Ensifer meliloti strains on Medicago sativa cv. Longzhong. E. meliloti strains are coded from 1 to 32 in the following order: G3L2, G3L3, G3L4, G3L5, G3L6, G3L7, G3L8, G3L9, G3L10, G3L12, G3L13, G3T2, G9L3, G9L4, G9L5, G9L6, G9L7, G9L8, LL1, LL2, LL5, LL6, LL7, LL8, LL10, LL11, LP3, QL2, QL4, QL5, WLG1 and WLP2. Red and green boxes indicate significant higher and lower values than the uninoculated control, respectively (P < 0.05). Strains subjected to transcriptome analysis are marked with blue lines and purple boxes (****, significance at P < 0.0001; ns, no significance at P < 0.05). Data are means and standard errors of four biological replicates. [file 12870_2020_2503_MOESM6_ESM.tif]

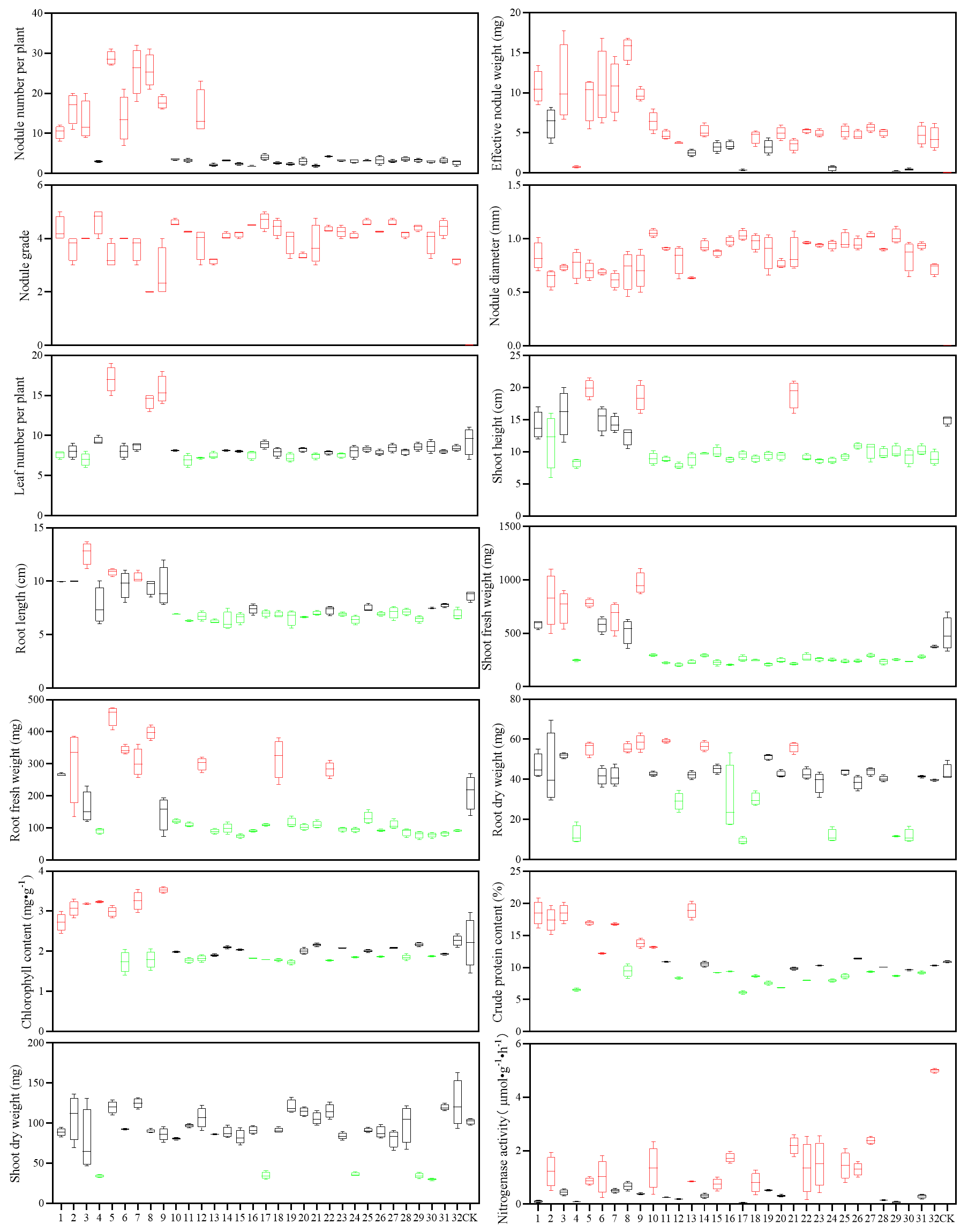

Supplement: Supplementary file 7 — Additional file 7. Symbiotic performance of 32 Ensifer meliloti strains on Medicago sativa cv. WL168HQ. E. meliloti strains are coded from 1 to 32 in the following order: G3L2, G3L3, G3L4, G3L5, G3L6, G3L7, G3L8, G3L9, G3L10, G3L12, G3L13, G3T2, G9L3, G9L4, G9L5, G9L6, G9L7, G9L8, LL1, LL2, LL5, LL6, LL7, LL8, LL10, LL11, LP3, QL2, QL4, QL5, WLG1 and WLP2. Red and green boxes indicate significant higher and lower values than the uninoculated control, respectively (P < 0.05). Strains subjected to transcriptome analysis are marked with blue lines and purple boxes (****, significance at P < 0.0001; ns, no significance at P < 0.05). Data are means and standard errors of four biological replicates. [file 12870_2020_2503_MOESM7_ESM.tif]

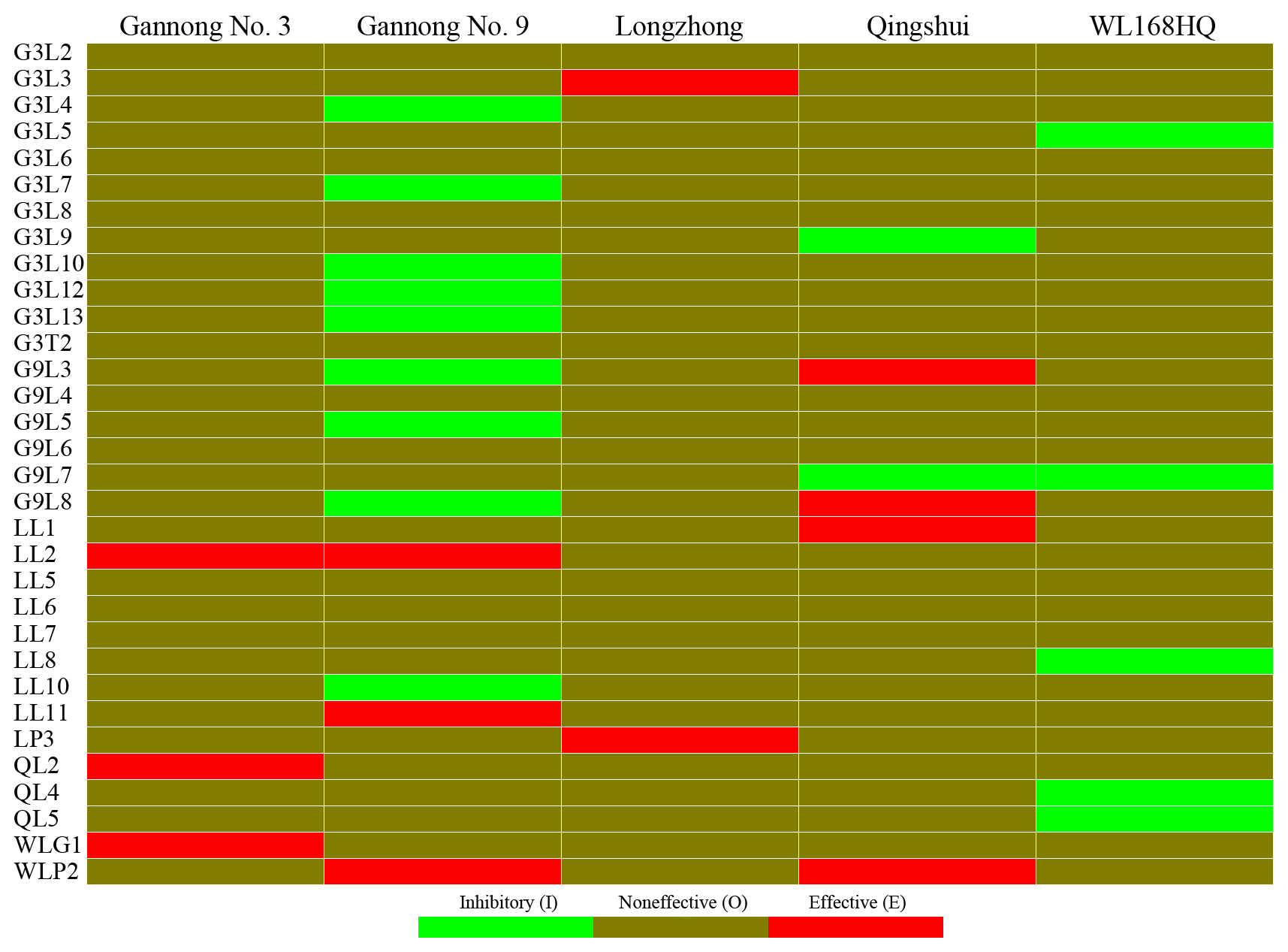

Supplement: Supplementary file 8 — Additional file 8. Assessment of the symbiotic efficiency for 32 Ensifer meliloti strains on five alfalfa cultivars. Strains were placed into the following three categories: effective (E), noneffective (O) and inhibitory (I) with shoot dry weight values significantly higher, no significant difference and significantly lower than that of the uninoculated control plants, respectively (P < 0.05). [file 12870_2020_2503_MOESM8_ESM.tif]

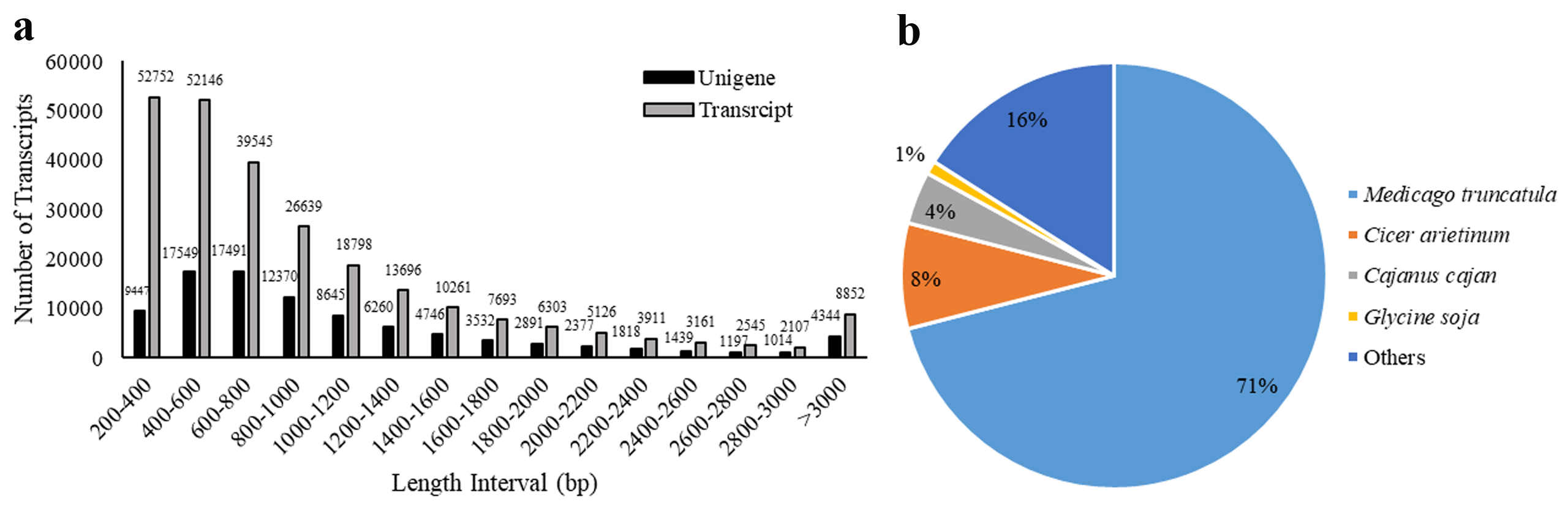

Supplement: Supplementary file 10 — Additional file 10. Length distribution of de novo assembly and taxonomic source of BLAST matches for Medicago sativa unigenes. a Length distribution of de novo assembly; b Taxonomic source of BLAST matches. [file 12870_2020_2503_MOESM10_ESM.tif]

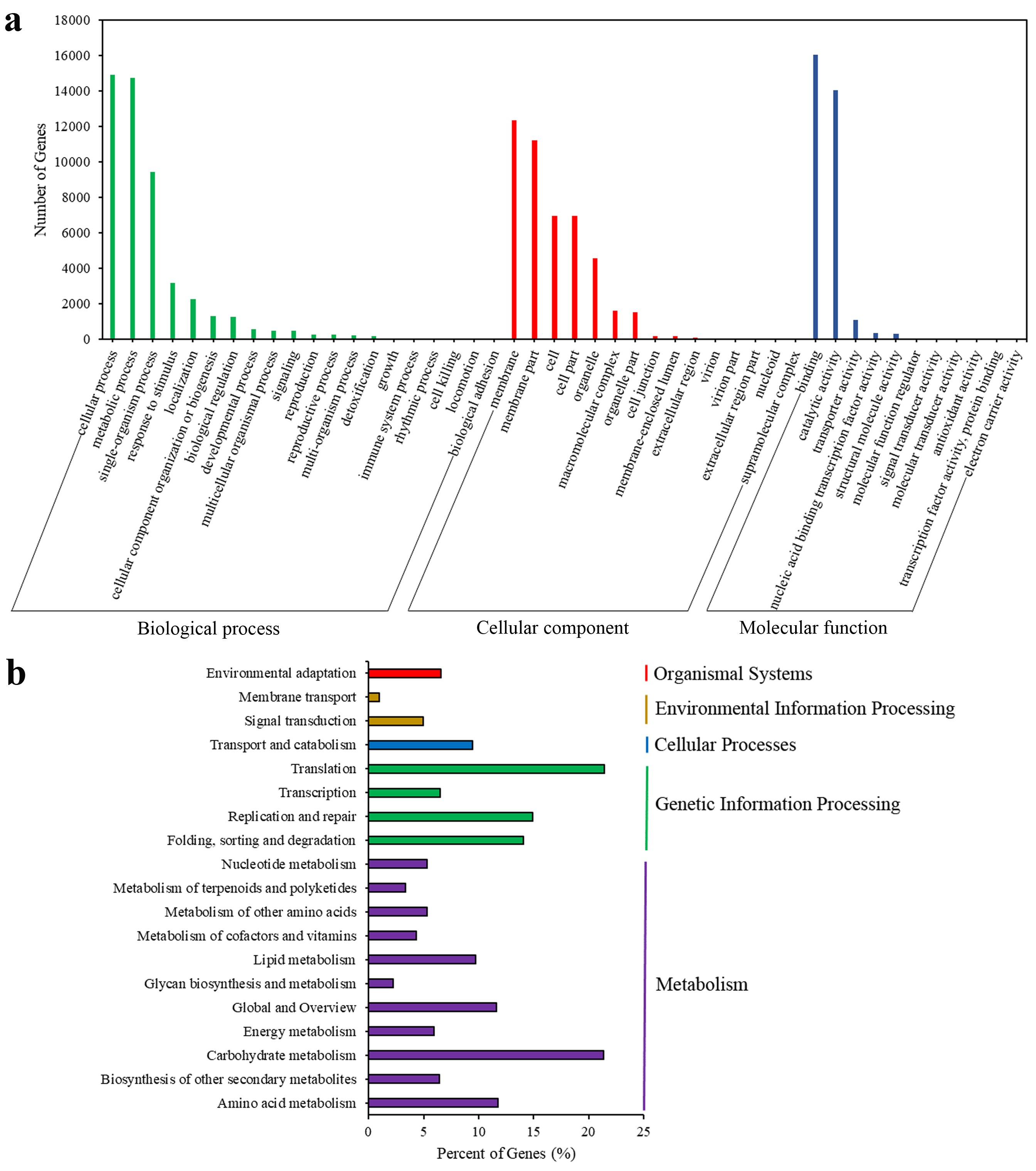

Supplement: Supplementary file 11 — Additional file 11. Gene ontology (GO) distributions and KEGG classification of the Medicago sativa transcriptomes. a The main functional categories in the biological process, cellular component and molecular functions categories found in the transcriptome. The ordinate indicates the number of unigenes. Bars represent the numbers of assignments of M. sativa proteins with BLASTx matches to each GO term. One unigene may be matched to multiple GO terms. b The left Y-axis indicates the KEGG pathway. Unigenes involved in metabolism pathways by KEGG classification are divided into five groups as shown on the right Y-axis. The X-axis indicates the percentage of unigenes that were assigned to a specific pathway. [file 12870_2020_2503_MOESM11_ESM.tif]

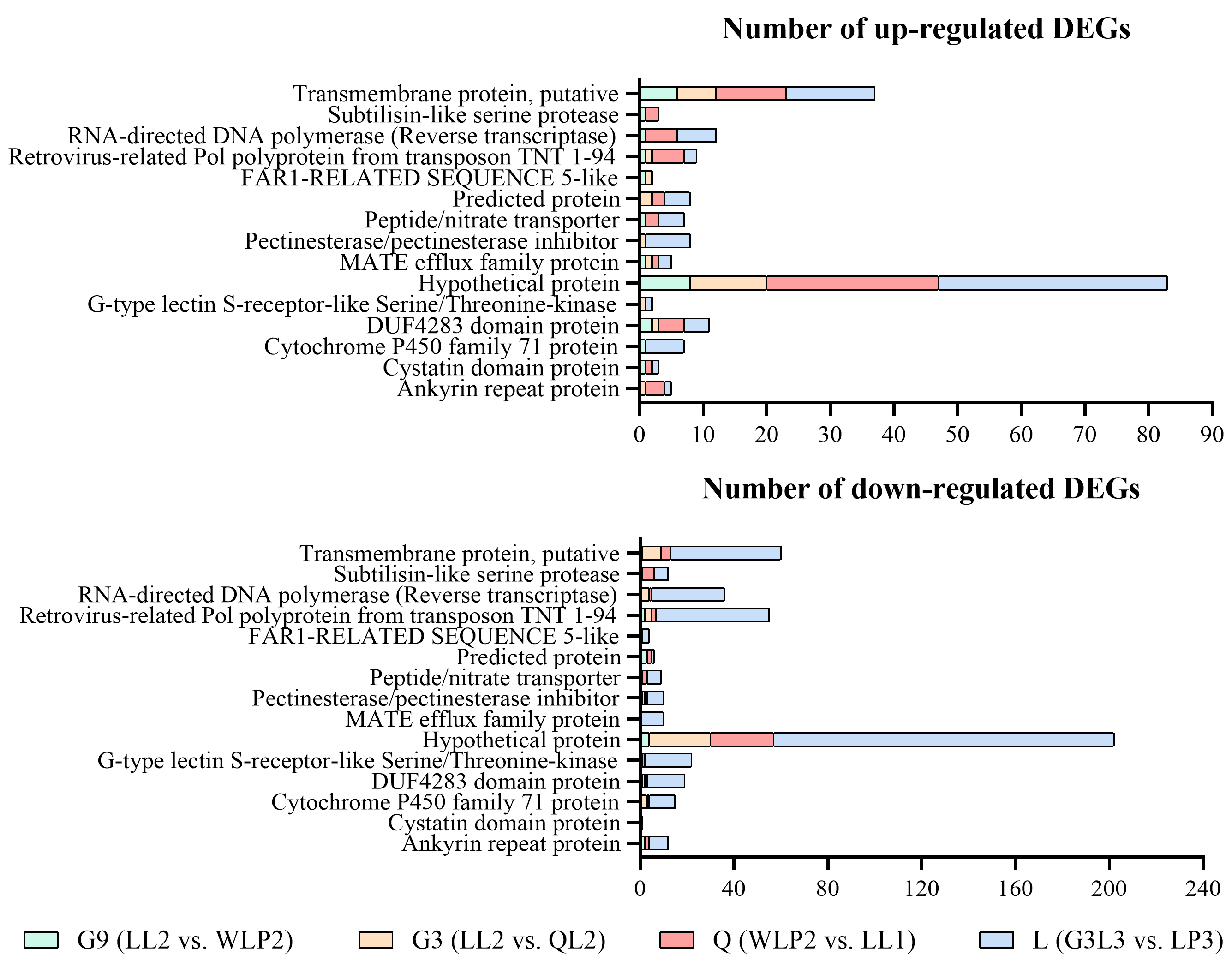

Supplement: Supplementary file 13 — Additional file 13. Numbers of up- and down-regulated DEGs commonly identified in four cultivars. Numbers of up- and down-regulated DEGs are shown for 15 proteins, except plant peptides and those involved in plant-pathogen interactions. [file 12870_2020_2503_MOESM13_ESM.tif]

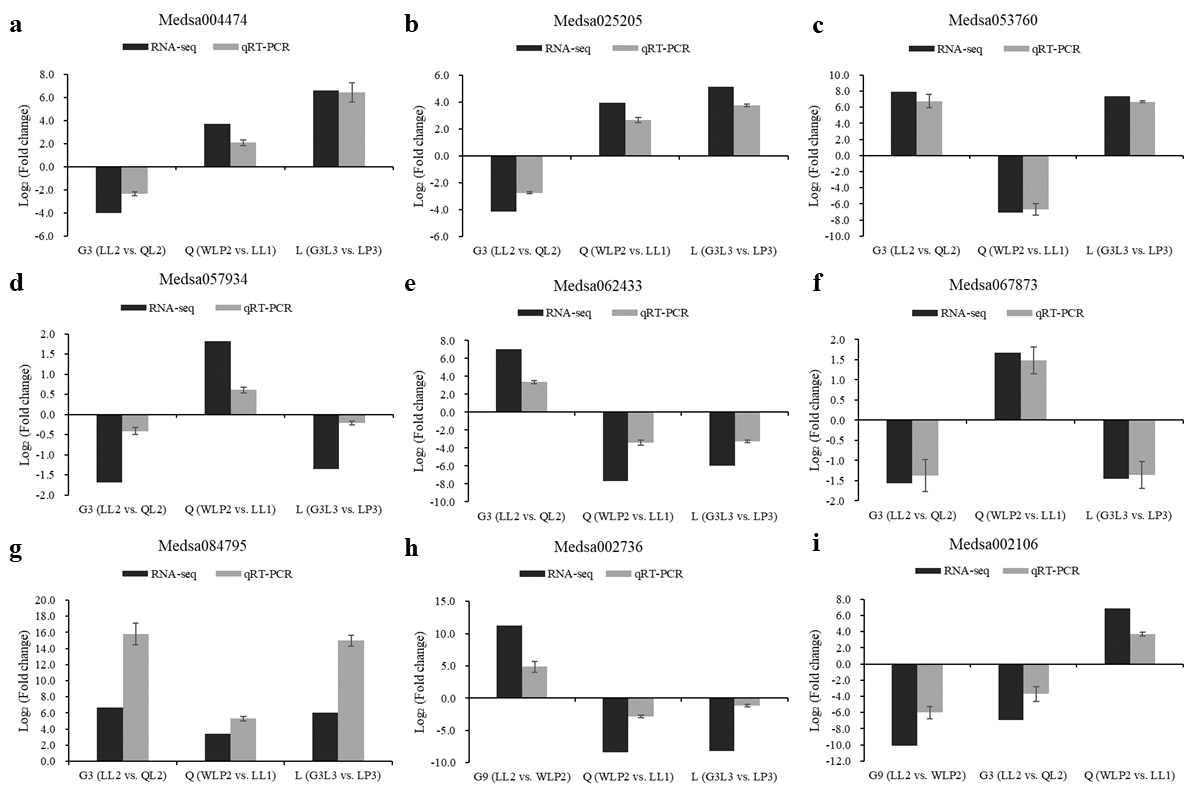

Supplement: Supplementary file 15 — Additional file 15. Validation of nine DEGs by Quantitative real-time PCR (qRT-PCR). The qRT-PCR data are means ± SEM of log2 (fold change) calculated using the 2-△△CT method. a-g seven genes jointly expressed in G3, Q and L. h one gene jointly expressed in G9, Q and L. i one gene jointly expressed in G9, G3 and L. [file 12870_2020_2503_MOESM15_ESM.tif]
