# Supplementary material for: Plant transcriptome analysis reveals specific molecular interactions between alfalfa and its rhizobial symbionts below the species level
Source: BMC Plant Biol. 2020 Jun 26;20:293. doi: 10.1186/s12870-020-02503-3 (PMC7318466; doi:10.1186/s12870-020-02503-3)
Supplement: Supplementary file 9 — Additional file 9. Summary of assembled transcriptome and function annotation of Medicago sativa. [file 12870_2020_2503_MOESM9_ESM.pdf]

Additional file 9: Summary of assembled transcriptome and function annotation of *Medicago sativa*

	Items	Number of bp/Reads/Transcripts
Sequencing and assembly	Total raw reads	1,923,792,442
	Total clean reads	1,913,483,492
	Total transcripts	253,535
	Total length of transcripts (bp)	247,565,414
	Transcripts with N50 length (bp)	1,310
	Transcript mean length (bp)	976
	Total unigenes	95,120
	Total length of unigenes (bp)	107,561,403
	Unigenes with N50 length (bp)	1,430
	Unigenes mean length (bp)	1,130
Function annotation	Nr database	67,815
	KEGG database	20,444
	Swissprot database	41,046
	KOG database	34,745
