# Supplementary material for: Plant transcriptome analysis reveals specific molecular interactions between alfalfa and its rhizobial symbionts below the species level
Source: BMC Plant Biol. 2020 Jun 26;20:293. doi: 10.1186/s12870-020-02503-3 (PMC7318466; doi:10.1186/s12870-020-02503-3)
Supplement: Supplementary file 12 — Additional file 12. Common genes expressed for an alfalfa cultivar inoculated with two rhizobial strains. [file 12870_2020_2503_MOESM12_ESM.pdf]

Additional file 12: Common genes expressed for an alfalfa cultivar inoculated with two rhizobial strains

Alfalfa cultivar	Comparison	Number of common genes	Ratio in *Medicago sativa* genome
G9	LL2 vs. CK<br>WLP2 vs. CK	4903	10.32%
G3	LL2 vs. CK<br>QL2 vs. CK	5294	11.14%
Q	WLP2 vs. CK<br>LL1 vs. CK	2824	5.94%
L	G3L3 vs. CK<br>LP3 vs. CK	1094	2.30%
All comparisons		110	0.23%
