# Supplementary material for: Plant transcriptome analysis reveals specific molecular interactions between alfalfa and its rhizobial symbionts below the species level
Source: BMC Plant Biol. 2020 Jun 26;20:293. doi: 10.1186/s12870-020-02503-3 (PMC7318466; doi:10.1186/s12870-020-02503-3)
Supplement: Supplementary file 14 — Additional file 14. Sequence of primers used in this work for quantitative real-time PCR (qRT-PCR). [file 12870_2020_2503_MOESM14_ESM.pdf]

Additional file 14: Sequence of primers used in this work for *q*RT-PCR

Genes	Primer sequences (5'-3') (forward/reverse)	Tm (℃)
Medsa004474	GCACCCACCACTACCTGTAGTC/CTGCCCAAACATTGAAAAGTATC	78.8
Medsa025205	AATTTCGTTCCTTCCACGATTC/TTTTCACGACCCTTGATTTCTAC	77.9
Medsa053760	CCCACTCAAGCTCTCGAAAAAG/ATAACGAATCTGGTTGGGACACT	80.9
Medsa057934	ACCAGGGAGAGGTCCTTTTACT/TAGCAAGGTAAAAGACAAACAACAG	79.1
Medsa062433	GGTCAATTCGGCACCACATATC/GAACCTCTCTCAGAACATCATCG	79.5
Medsa067873	AAACATCAAAGCAATGGAGCAC/AAACATCAAAGCAATGGAGCAC	77.2
Medsa084795	CAACGACGGTGGACTTCCTG/CTTGTCTCGCTCTCTCGCACT	83.1
Medsa002736	GAGCAAGGTGTACAAGTTTCAGAG/GTTGTCTCCTGAGCCACTTGTTC	79.2
Medsa002106	GATGAAAGCAAAGAGTGTGGAAT/TCCCTCGCTACTTTCACCAAC	79.9
β-actin2	AAGTCCAAAATGATGCGATAATG/CCTCCGATCTCACCTCTTATCC	78.5
